# Principles for Object-Linguistic Consequence: from Logical to Irreflexive

**DOI:** 10.1007/s10992-017-9438-x

**Published:** 2017-06-20

**Authors:** Carlo Nicolai, Lorenzo Rossi

**Affiliations:** 1 Munich Center for Mathematical Philosophy, Ludwig-Maximilians-Universität München, Munich, Germany; 2 Department of Philosophy (KGW), Universität Salzburg, Salzburg, Austria

**Keywords:** Object-linguistic consequence, V-Curry paradox, Logical consequence, Irreflexive consequence

## Abstract

We discuss the principles for a primitive, object-linguistic notion of consequence proposed by (Beall and Murzi, *Journal of Philosophy*, **3** pp. 143–65 (2013)) that yield a version of Curry’s paradox. We propose and study several strategies to weaken these principles and overcome paradox: all these strategies are based on the intuition that the object-linguistic consequence predicate internalizes whichever meta-linguistic notion of consequence we accept in the first place. To these solutions will correspond different conceptions of consequence. In one possible reading of these principles, they give rise to a notion of logical consequence: we study the corresponding theory of validity (and some of its variants) by showing that it is conservative over a wide range of base theories: this result is achieved via a well-behaved form of local reduction. The theory of logical consequence is based on a restriction of the introduction rule for the consequence predicate. To unrestrictedly maintain this principle, we develop a conception of object-linguistic consequence, which we call *grounded* consequence, that displays a restriction of the structural rule of *reflexivity*. This construction is obtained by generalizing Saul Kripke’s inductive theory of truth (strong Kleene version). Grounded validity will be shown to satisfy several desirable principles for a naïve, self-applicable notion of consequence.

## Introduction

Object-linguistic treatments of *consequence* have been extensively investigated in the recent literature: on these approaches, consequence is formalized as a predicate in some first-order language, and principles governing its behaviour are given. These studies are motivated by diverse philosophical aims, ranging from criticisms to paraconsistent theories [39], to deflationism about consequence [34], to new versions of truth-theoretical paradoxes such as Curry’s paradox [1, 18, 27]. Some of these authors, Beall and Murzi [1] and Murzi and Shapiro [23] in particular, also stress the analogy between object-linguistic treatments of consequence, truth, and comprehension, and call for a unified solution of the resulting paradoxes, arguing that substructural approaches are preferable to fully structural ones.^1^ In order to conform with the terminology adopted in the literature, we will treat ‘consequence’ and ‘validity’ as synonymous where, crucially, consequence or validity do not necessarily coincide with logical consequence or logical validity.

All these approaches can in fact be seen as investigating different ways in which some conclusion ‘follows from’ some premises. In their recent [1], Beall and Murzi proposed the following *naïve* principles for a primitive validity predicate *Val*(*x*, *y*): 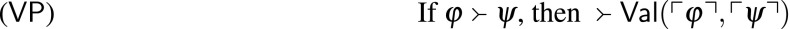

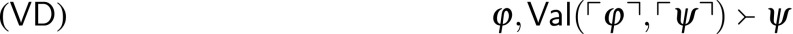
 where *φ* and *ψ* range over sentences possibly containing *Val* itself, and $\ulcorner \cdot \urcorner $ is informally understood as a name-forming device. It is not completely clear how to read *⤙* : Beall and Murzi [1] interpret it as an unspecified relation of ‘following from’. They also introduce (what they see as) an analogue of the disquotation schema for truth: 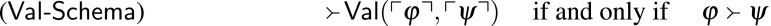


(*VP*) and (*VD*) are inconsistent with classical logic, over a sufficiently expressive base theory. Beall and Murzi show this via a variant of Curry’s Paradox, which they call *V-Curry Paradox*. In order to introduce the paradox, let us fix the meaning of *⤙* as a sequent arrow of a system including (*VD*) and the axioms of a sufficiently strong syntax theory as initial sequents, and closed under (*VP*) and the standard logical rules – notably, left contraction and cut. Our syntactic axioms enable us to find a sentence *ν* that is inter-derivable with $\mathsf {Val}(\ulcorner \nu \urcorner ,\ulcorner \bot \urcorner )$, where ⊥ is some falsity of the base theory. Reasoning in the naïve theory of validity, we have: 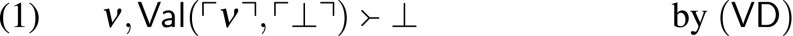

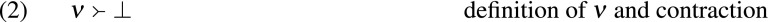

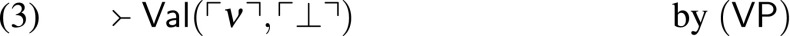

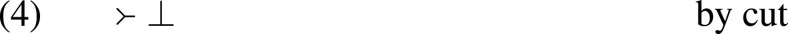


In this paper, we explore different strategies to block the V-Curry and related paradoxes. These strategies fall under a common intuition: starting with some meta-theoretic consequence relations, we internalize them in the object language in ways that capture their fundamental traits. That is, each such strategy corresponds to the acceptance of different principles for *Val* and to different restrictions of the structural rules. Different solutions to the paradox, then, correspond to different ways of cashing out the idea that the acceptance of a sentence of the form $\mathsf {Val}(\ulcorner \varphi \urcorner ,\ulcorner \psi \urcorner )$ is ultimately to be explained and justified by the acceptance of some meta-theoretical validity statements, where the acceptance of the latter does not involve object-linguistic validity principles.

A natural option to develop this strategy, and block the V-Curry (and related paradoxes), is to apply (*VP*)*only to logical derivations*. Under this reading, *Val* becomes a primitive predicate for logical validity. This is the strategy followed by Ketland [15]: he axiomatizes *Val* over Peano Arithmetic (henceforth *PA*) and proves the consistency of the theory resulting from this restriction of (*VP*). This option is supported by the fact that (*VP*) does not preserve *logical* validity. The very possibility of formulating (*VP*) requires a well-behaved machinery to handle the name-forming device $\ulcorner \cdot \urcorner $ and this machinery does not satisfy uniform substitutivity, violating a basic requirement for logically valid principles.^2^

However, Ketland’s consistency proof only applies to a restricted category of theories. These theories will be called later reflexive theories. Moreover, Ketland’s strategy is based on the possibility of reducing the primitive logical validity predicate to a provability predicate definable in *PA*. Besides establishing consistency, such proof-theoretic reductions (such as conservativity and variants of interpretability) also help us in characterizing the notion associated with the logical validity predicate. For instance, the reducibility of the truth predicate to the base theory may be used to assess general conceptions of truth such as truth-theoretical deflationism (see [14, Ch. 7]): in the same way, proof-theoretical reductions might be employed to assess deflationary and other general conceptions of logical validity (see [34]). Therefore, in Section 2, we provide more general reduction techniques that, besides yielding a consistency proof, will give us a finer-grained analysis of logical validity axiomatized over a wide range of base theories.^3^

A noteworthy feature of object-linguistic logical validity is that *iterations* of the validity predicate are not allowed. For if *φ* *⤙* *ψ* is logically valid we can conclude $\mathsf {Val}(\ulcorner \varphi \urcorner , \ulcorner \psi \urcorner )$ in the theory of logical validity, but from the latter we cannot conclude $\mathsf {Val}(\ulcorner \varnothing \urcorner , \ulcorner \mathsf {Val} (\ulcorner \varphi \urcorner , \ulcorner \psi \urcorner ) \urcorner )$. There are, however, different notions of consequence, and notions expressing ‘following from’ more generally, for which iterations are very natural, such as entailment or implication.^4^ A standard option to approximate iterability is resorting to *hierarchies*, namely stratifying the *⤙* and the validity predicate. For example, Field [9] suggests a hierarchy of validity predicates and sequent arrows, and the following version of Beall and Murzi’s principles, where *⤙*_*β*_ is read as ‘derivability in the theory of validity of level *β*’: 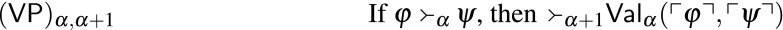

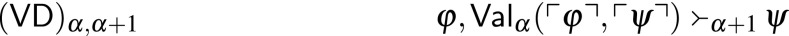


In Section 2.4 we will see that this stratified notion of validity may be understood in terms of a hierarchy of *reflection principles* over the starting theory: therefore, not only stratified object-linguistic validity is classically consistent, but it has a natural conceptual analysis in terms of a hierarchy of soundness extensions of the starting theory.

However, like any hierarchical approach, also this proposal suffers from variants of the so-called ‘Nixon-Dean problem’ (see [16], pp. 694–697); consider for example the following case

Speaker Asays: ‘the negation of everything Isay follows from what Speaker Bsays’.

Speaker Bsays: ‘everything Isay follows from what Speaker Asays’.

As for truth, these cases pose problems for hierarchical and non-self-applicable accounts of consequence.

In Sections 3–4, we develop an approach to object-linguistic validity that overcomes this problem: it blocks paradoxical arguments and, at the same time, avoids restrictions on (*VP*), recovers a natural version of (*VD*), and delivers a single and genuinely self-applicable notion of validity. This will be accomplished by an inductive construction that generalizes the one in [16] (strong Kleene version). The fundamental feature of the construction is that the models (for languages with self-applicable validity) it generates do not satisfy the structural rule of reflexivity.^5^ The smallest fixed point of our construction yields a notion that we might call *grounded validity*, in that it extends Kripke’s notion of grounded truth (see [16], pp. 694 and 706–707). This is because the meta-theoretical notion of validity that holds in the base language determines the extension of the object-linguistic validity predicate. As we argue in Section 5, grounded validity affords us a natural reading of the naïve validity-theoretical principles.

## Object-Linguistic Validity and Classical Logic

One may be tempted to read the rules (*VP*) and (*VD*) as characterizing a notion of logical validity or logical consequence. However, it became soon clear that this temptation should be resisted: object-linguistic treatments of logical consequence simply do not give rise to paradox. This is the conclusion reached by Cook [7] and Ketland [15], and echoed by Field [9]. In particular, the former analyze the Curry-like derivation sketched in the introduction and come to the main conclusion that paradox arises when the principles governing this notion (whatever it may be) of primitive consequence are themselves considered to be logically valid.^6^

In the following two subsections we consider strategies to overcome paradox while keeping classical logic. But we do not only aim at (classical) consistency: by considering suitable reduction methods of the theories with primitive validity to the respective base theory or suitable extensions of it, we intend to study the nature of the concept of validity in relation with the inferential resources of the starting base theory. In particular:

1. Improving on [7, 15], we give a uniform method for the conservativity of the theories of logical validity over an arbitrary theory extending Elementary Arithmetic (*EA*), a very weak arithmetical theory.
2. This method will also yield the reducibility of the theory of logical validity to reflexive base theories (e.g. *PA* as in [7, 15]) and its local reducibility in finitely axiomatized based theories (e.g. *EA* itself), in which ‘reduction’ is intended as a well-behaved version of relative interpretability that preserves arithmetical vocabulary.
3. Even if logical validity is extended to purely arithmetical consequence, classical logic can consistently be kept by interpreting *Val* as provability in the base theory. Starting form this observation, we show that the hierarchy of validity predicates hinted at by Field [9] can be naturally interpreted as a hierarchy of local reflection principles for the starting theory.

These formal results suggest in turn that the notions of primitive logical and arithmetical (or syntactic) validity are not only unparadoxical, but that they can be conceptually reduced, either globally or locally, to notions definable in the base theory or extensions in the same language.

### Arithmetical Theories and Reductions

We now fix some formal details. We work in the language $\mathcal {L}=\{0,\mathsf {S},+,\times ,\mathsf {exp},$ ≤} of arithmetic. Occurrences of the quantifiers in expressions of the form (∀*x* ≤ *t*) *φ*(*x*) and (∃*x* ≤ *t*) *φ*(*x*) where *t* does not contain *x* are called *bounded*. Formulas containing only bounded occurrences of quantifiers are called *elementary formulas* or Δ_0_-*formulas*.

All theories considered below will extend Elementary Arithmetic *EA* (or, equivalently, *I*Δ_0_ plus the totality of exponentiation).^7^

The class of *elementary functions* $\mathcal {E}$ is obtained by closing the initial functions *zero*(⋅), **S***uc*(⋅), + , ×, 2^*x*^, $\mathsf {P}_{i}^{n}(x_{1},...,x_{n})=x_{i}$ with (1 ≤ *i* ≤ *n*), and truncated subtraction $x\dot -y$ under the operations of composition and bounded minimalization:

$$H(\mathbf{x})\,=\,F(G_{1}(\mathbf{x}),\ldots,G_{n}(\mathbf{x})); \quad\quad (\mu t\leq y)\;P(\textbf{x},t)\,=\,\left\{\begin{array}{l}\,\text{the least}\, t\leq y\, \text{s.t.}\, P(\mathbf{x},t)\\ \,0, \, \, \, \text{if there is no such}\, t \end{array}\right. $$

where *F*, *G*_1_,…,*G*_*n*_ are elementary functions and *P* an elementary predicate. *EA* has sufficient resources to naturally introduce new relations corresponding to the elementary functions by proving their defining equations. We will therefore freely employ some functional expressions for the relevant elementary operations and relations.

The formalization of the syntax of first-order theories as it is standardly done in, e.g., [33], is carried out without difficulties in *EA*. In particular, once we show that the standard arithmetization of the syntax can be captured by elementary functions, the fact that *EA* can Σ_1_-define precisely the elementary functions ensures us that syntactic predicates and notions can be intensionally captured in it.

Unless otherwise specified, throughout this section we fix a Hilbert-style system for first-order logic in which modus ponens is the only rule of inference: $X\vdash \varphi $ then indicates that there is a derivation in this system of *φ* from sentences in *X*, logical axioms, and using modus ponens only. Derivations will therefore be sequences of formulas. Also the syntactic notion of *relative interpretation* of a theory *U* presented via an elementary set of axioms into another elementary presented theory *W* will repeatedly occur:^8^ it can be considered as a triple (*U*, *τ*, *W*), with *τ* a translation function $\tau \colon \mathcal {L}_{U}\to \mathcal {L}_{W}$ that maps *n*-ary relations of $\mathcal {L}_{U}$ into $\mathcal {L}_{W}$-formulas with *n* free variables, *n*-ary functions of $\mathcal {L}_{U}$ into $\mathcal {L}_{W}$-formulas with *n* + 1 free variables satisfying the obvious existence and uniqueness conditions, and that relativizes quantifiers to a suitable $\mathcal {L}_{W}$-formula *δ*(*x*), the domain of the translation. In addition, (*U*, *τ*, *W*) satisfies

$$\text{if }\;U\vdash \varphi,\;\text{ then }\;W\vdash \bigwedge_{x_{i}\in \mathsf{FV}(\varphi)}\delta(x_{i})\rightarrow \varphi^{\tau} $$

for formulas $\varphi \in \mathcal {L}_{U}$ and *FV*(*φ*) the set of free variables of *φ*.

A relative interpretation preserves the structure of a proof. On many occasions we will employ a more regimented notion of relative interpretation. An interpretation is direct if quantifiers are unrelativized and identity is mapped into identity. Let *U* and *W* be such that $\mathcal {L}\subseteq \mathcal {L}_{U}\cap \mathcal {L}_{W}$. We say that *U* is $\mathcal {L}$ *-embeddable* in *W* if there is a direct interpretation of *U* into *W* that leaves the $\mathcal {L}$-vocabulary unchanged. $\mathcal {L}$-embedding is a properly stricter notion than relative interpretability.^9^ Finally, *U* is *locally interpretable* in *W* if any finite subtheory of *U* is relatively interpretable in *W*. The notion of *local* $\mathcal {L}$ *-embedding* is defined analogously.

### Object-Linguistic Logical Validity

As anticipated, in this subsection we deal with object-linguistic treatments of *logical* validity: that is we focus on theories that will be obtained by restricting (*VP*) only to purely logical derivations. It is worth remarking here that, since we are employing classical logic, the deduction theorem holds: this makes the presentation of the theories of logical validity smoother.

#### **Definition 1**

*Let* $T\supseteq \mathsf {EA}$ *be a consistent theory formulated in* $\mathcal {L}_{V}=\mathcal {L} \cup \{\mathsf {Val}\}$. *The theory* *T*^*V*^_0_*extends* T *with the following principles*, *for all* $\mathcal {L}_{V}$ *-sentences* *φ*, *ψ*: 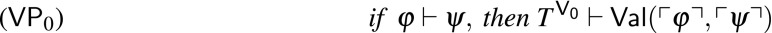

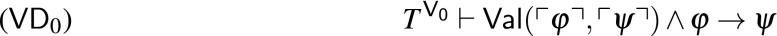
 *We refer to the theory* ${ T}^{\mathsf {V_{0}}}\mathpunct \upharpoonright $ *as the theory obtained from* *T*^*V*^_0_ *by allowing only formulas of* $\mathcal {L}$ *as instances of nonlogical axiom schemata of* *T*.

*T*^*V*^_0_ results from restricting (*VP*) to purely logical derivations. However, since conditional introduction will be assumed throughout this section, it is convenient to work with a unary rather than a binary validity predicate.

#### **Definition 2** (Primitive logical validity)

*Let* $T\supseteq \mathsf {EA}$ *be a consistent theory formulated in* $\mathcal {L}^{+}=\mathcal {L}\cup \{\mathsf {V} \}$, *where* *Vis now intended as a unary predicate. The theory* *T*^*V*^*extends* T *with the principles*, *for all* $\mathcal {L}^+$ *-sentences* *φ*: 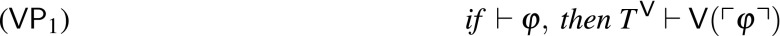

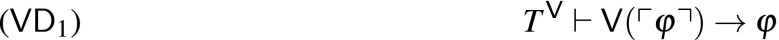
 *Again we refer to the theory* ${ T}^{\mathsf {V}}\mathpunct \upharpoonright $ *as the theory obtained from* *T*^*V*^ *by allowing only formulas of* $\mathcal {L}$ *as instances of nonlogical axiom schemata of* *T*.

That *T*^*V*^ is no essential modification of *T*^*V*^_0_ is guaranteed by the following:

#### **Proposition 1**

*T*^*V*^*and* *T*^*V*^_0_*are mutually* $\mathcal {L}$ *-embeddable*, *and so are* $T^{\mathsf {V}}\!\!\upharpoonright $ *and* $T^{\mathsf {V_{0}}}\mathpunct \upharpoonright $.

#### *Proof*

The idea is entirely straightforward. By employing the recursion theorem to translate within Gödel quotes,^10^ we can uniformly replace *Val*(*x*, *y*)and *V*(*x*)with, respectively, $\mathsf {V}(\tau _{0}(x\underset {\cdot }{\rightarrow } y))$ and $\mathsf {Val}(\ulcorner 0=0\urcorner ,\tau _{1} (x))$ where *τ*_0_, *τ*_1_ are suitable (elementary) translations that leave the arithmetical vocabulary unchanged and do not relativize quantifiers. The verification that the two translations are in fact$\mathcal {L}$-embeddingsis routine as the following holds, for *i* ≤ 1and *φ* either in $\mathcal {L}^{+}$ or in $\mathcal {L}_{V}$:

$$(5)~~~~~~~~~~~~~~~~~~~~~~~~~~~~~~~~~~~\text{if}\, \varphi\, \text{is provable in pure logic, then so is}\, \varphi^{\tau_{i}}{\kern6pc} $$

□

It is intuitively fairly clear that the derivation of the V-Curry paradox is blocked in *T*^*V*^. When (*VP*) is applied in the informal presentation of the paradox on p. 2, (*VD*) has been already employed and therefore (*VP*), in the step between (3) and (4), cannot be applied, since the sequent in (3) is not obtained via a purely logical derivation. This indicates a strategy to prove the consistency of *T*^*V*^, which anticipates some traits of the construction carried out in Section 3.2. The following is a positive inductive definition of the set *S* of logical truths of $\mathcal {L}^+$:

$$(6)~~~~~~~~~~~~y\in S\,\leftrightarrow\,\mathsf{Sent}_{\mathcal{L}^+}(y)\land \left(\mathsf{LAx}(y)\vee \exists x\,(x \in S \land (x\underset{\cdot}{\rightarrow} y)\in S)\right){\kern3pc} $$

where $\mathsf {Sent}_{\mathcal {L}^+}$ and *LAx* are elementary predicates representing the set of (codes of) sentences of $\mathcal {L}^{+}$ and of logical axioms respectively. A fixed point of this definition is a set *X* such that $(\mathbb {N},X)$ models (6) in the sense that *X* is taken as the extension of *S*. It is easy to see that the least such fixed point *I*_*V*_ is reached after *ω* iterations of the operator associated with (6) and that the structure $(\mathbb {N},\mathsf {I}_{V})$ is a model of *T*^*V*^ (and $T^{\mathsf {V}}\!\!\upharpoonright $).

However, consistency may be seen as a necessary but not sufficient condition for a full characterization of the concept of validity captured by *T*^*V*^. As we mentioned above, a primitive validity predicate is usually motivated – for instance in [1, 23, 34] – along similar lines as the truth predicate: in both cases we aim at expressing meta-theoretic facts in the object-language. For instance, one might want to prove in *T*^*V*^ that all tautologies are logically valid, or that so are all implications from a finite subsets of the axioms of *T*^*V*^. A natural question concerns therefore the costs of the extra expressive power given by *V* with respect to the inferential resources of the base theory. Moreover, it is mathematically interesting to weigh these costs across a wide range of possible syntactic base theories by abstracting away from specific conditions related to a particular choice of the base theory. From this point of view, a general study of the properties of the theory of object-linguistic validity such as its $\mathcal {L}$-embedding in the base theory *T*, conservativity over *T*, finite axiomatizability over *T*, become integral part of the study of this notion of validity.

The analogy with truth cannot be pushed much further; in particular, it would be a mistake to see theories of logical validity as a subspecies of theories of truth. Theories of truth featuring the truth-theoretic version of (*VD*_1_) are usually prone to an asymmetry between the internal theory – i. e. what the theory proves true – and the set of its theorems: they prove the conjunction $\lambda \land \neg \mathsf {T}\ulcorner \lambda \urcorner $ for some sentence *λ*, where *T* is the truth predicate. In other words, the theory displays the puzzling feature of asserting a sentence while declaring it untrue.^11^ The situation in *T*^*V*^ is both similar and radically different. By diagonalization, we can obviously obtain a sentence *χ* such that

$$(7)~~~~~~~~~~~~~~~~~~~~~~~~~~~~~~~~~~~~~~~~~~~~~~{T^{\mathsf{V}}} \vdash \chi\leftrightarrow \neg \mathsf{V}(\ulcorner \chi\urcorner){\kern10pc} $$

By (*VD*_1_) and (7), we can derive $\neg \mathsf {V} (\ulcorner \chi \urcorner )$ and therefore *χ* in *T*^*V*^. However, if *V* is interpreted as logical validity, it is not only harmless, but even desirable for *χ* to be derivable in *T*^*V*^ but not logically valid because its derivation crucially involves (*VD*_1_).

Next we show that the primitive notion of consequence given by *T*^*V*^ cannot serve an expressive role of finite re-axiomatization.^12^

#### **Lemma 1**

*T* ^*V*^ *is not finitely axiomatizable.*

#### *Proof*

Seeking a contradiction, let *T*_0_ be finite reaxiomatization of *T*^*V*^ such that $T_{0} \dashv \vdash {T^{\mathsf {V}}} $. This entails that we can find a finite subtheory **A** *of* *T*^*V*^ such that $\mathsf {A} \dashv \vdash {T^{\mathsf {V}}} $ and in which (VP_1_) and (VD_1_) can only be applied to sentences of $\mathcal {L}^{+}$ containing at most *n* logical symbols. Let $\mathcal {L}^{+}_{n}$ be this latter set of sentences. Now adapt (6) in the following way, where $\mathsf { Sent}_{\mathcal {L}_{n}^{+}}$ is the set of (codes of) sentences of $\mathcal {L}^{+}$ containing at most *n* occurrences of logical symbols:

$$\begin{array}{@{}rcl@{}} k\in S\;\leftrightarrow \;k&=&\ulcorner \neg(\underbrace{\top\land\ldots\land \top}_{\land\, \text{applied}\, n\text{-times}})\urcorner \;\vee\\ &&{}\left[k\!\in\! \mathsf{Sent}_{\mathcal{L}_{n}^{+}}\!\land\! \left(\mathsf{LAx}(k)\!\vee\! \exists m\,(m\in \mathsf{Sent}_{\mathcal{L}_{n}^{+}}\!\land\! m \in S \!\land\! (m\underset{\cdot}{\!\rightarrow\!} k)\!\in\! S)\right)\right] \end{array} $$

Let *I*_*V*_^*n*^ be the least fixed point of this inductive definition. $(\mathbb {N},\mathsf {I}_{V^{n}})$ is a model of **A** but it cannot be a model of *T*^*V*^. □

We now move on to the question of the conservativity and $\mathcal {L}$-embeddability of *T*^*V*^ in *T*. In what follows, we distinguish between *reflexive* and *finitely axiomatizable* extensions of **EA**. We recall that a theory is *reflexive* if it proves the consistency of any of its finite subtheories: for all natural choices of *T*, reflexive theories *T* prove, for finite $S\subset T$ and for all $\varphi \in \mathcal {L}$,

$$(\textsf{Rfn}(S))~~~~~~~~~~~~~~~~~~~~~~~~~~~~~~~~~~~~~~~~~~~~~~~~~~~~~~~~\mathsf{Pr}_{S}(\ulcorner \varphi\urcorner)\rightarrow \varphi{\kern20pc} $$

where *Pr*_*S*_(⋅) is a canonical provability predicate for *S*.

For reflexive *T*, the question of the $\mathcal {L}$-embedding and conservativity of *T*^*V*^ in *T* is readily obtained: let us define the elementary translation $\mathfrak {a}\colon \mathcal {L}^{+}\to \mathcal {L}$:

$$\begin{array}{@{}rcl@{}} &&(s=t)^{\mathfrak{a}}:= s=t (\mathsf{V} t)^{\mathfrak{a}}:=\mathsf{Pr}_{\varnothing} (\mathfrak{a}(t))\\ &&(\neg \varphi)^{\mathfrak{a}}:=\neg \varphi^{\mathfrak{a}} (\varphi\land \psi)^{\mathfrak{a}}:=\varphi^{\mathfrak{a}}\land \psi^{\mathfrak{a}}\\ &&(\forall x\varphi)^{\mathfrak{a}}:=\forall x \varphi^{\mathfrak{a}} \end{array} $$

The definition of $\mathfrak {a}$ again relies on Kleene’s recursion theorem: in particular $\mathfrak {a}(\cdot )$ represents $(\cdot )^{\mathfrak {a}}$ in **EA**; moreover, $\mathsf {Pr}_{\varnothing }(\cdot )$ stands for canonical logical provability, that is provability from the empty set of nonlogical assumptions.

#### **Lemma 2**

(i) *If T* *is reflexive*, *then* $\mathfrak {a}$ *is an* $\mathcal {L}$ *-embeddingof* *T*^*V*^*in T*.
(ii) *If* *T is finitely axiomatizable*, $\mathfrak {a}$ *cannot be an interpretation of* *T*^*V*^*in T*.

#### *Proof*

Both proofs are immediate. For (i), one simply notices that *T*, being reflexive, proves $\mathsf {Pr}_{\varnothing }(\ulcorner \varphi \urcorner )\rightarrow \varphi $ for all $\varphi \in \mathcal {L}$ by Rfn(*S*).

For (ii), if $\mathfrak {a}$ were an interpretation of *T*^*V*^ in *T*, by letting **A** be again a finite axiomatization of *T*, we would have

$$\mathsf{A}\vdash \mathsf{Pr}_{\mathsf{A}}(\bot )\rightarrow \bot, $$

(with$\bot :=\ulcorner 0=1\urcorner )$,which contradicts Gödel’s second incompleteness theorem. □

Part (i) of the previous lemma obviously entails the interpretability of *T*^*V*^ in *T* for reflexive *T*, being $\mathcal {L}$-embeddings stricter than intepretability. Two further remarks: the interpretation $\mathfrak {a}$ is a variant of the one contained in [15], which only takes care of external occurrences of *V* without applications of the recursion theorem. Moreover, in Lemma 2(i), $\mathfrak {a}$ is indeed an $\mathcal {L}$-embedding of *T*^*V*^ in *T* when the latter is reflexive. This clearly indicates that, in the case of reflexive theories, the notion of validity governed by (VP_1_) and (VD_1_) can be unequivocally understood as a definable notion of logical validity. Lemma 2(i) also immediately yields the conservativity of *T*^*V*^ over *T* for reflexive *T*.

However reflexive theories are in many senses very special and they have a peculiar behaviour with respect to interpretability and related notions. For instance, by Orey’s compactness theorem,^13^ reflexive theories collapse the distinction between local and global interpretability and they have the very convenient feature of proving the reflection principle for pure logic that is, as we have seen, closely related to (VD_1_). It is therefore natural to generalize the picture given by Lemma 2 and ask ourselves whether the notion of consequence captured by (VP_1_) and (VD_1_) can be uniformly characterized also in the case of non-reflexive theories. As we anticipated, we will focus on finitely axiomatized theories, which are provably distinct from reflexive theories due to Gödel’s second incompleteness theorem.

We recall that *U* is *locally interpretable* in *V* if every finite subtheory of *U* is interpretable in *V*. Similarly, *U* is *locally* $\mathcal {L}$ *-embeddable* in *V* if every finite $U_{0}\subseteq U$ is $\mathcal {L}$-embeddable in *V*. We also recall that *T*^*V*^ is formulated in a Hilbert-style calculus in which modus ponens is the only rule of inference. Proofs in *T*^*V*^ of *φ* are therefore objects of the form $ \mathcal {D}=\langle \varphi _{0},\ldots ,\varphi _{n-1},\varphi \rangle $ where each element of the sequence is either an axiom of *T*^*V*^ or it has been obtained from previous elements by modus ponens. Also, the code $|\mathcal {D}|$ of $\mathcal {D}$ is the code of $\langle \ulcorner \varphi _{0}\urcorner ,\ldots ,\ulcorner \varphi _{n-1}\urcorner \rangle $.

To prove the local $\mathcal {L}$-embeddability of *T*^*V*^ in *T*, we need the following, well known fact:

#### **Lemma 3** (Σ_1_-completeness)

*For every* Σ_1_*-formula* *φof* $\mathcal {L}$ *and every* T *extending* **EA**, *if* $\mathbb {N}\vDash \varphi $, *then* $\, T\vdash \varphi $.

The informal idea for the proof of the local $\mathcal {L}$-embeddability of *T*^*V*^ in *T* is straightforward: we translate only the outermost occurrences of *V* because only one ‘layer’ of the logical validity predicate matters in logical proofs. This enables us to dispense with uses of more sophisticated devices, such as the recursion theorem, to translate within Gödel corners.

#### **Proposition 2**

*T*^*V*^*is locally* $\mathcal {L}$ *-embeddablein* T.

#### *Proof*

Let *B* be a finite subsystem of *T*^*V*^. In *B*, we can safely assume that there are at most *m* applications of (VP_1_) to logical proofs $\mathcal {D}_{i}$, *i* ≤ *m*.

Let $\,|\mathcal {D}_{i}|\,\leq n$ for all logical proofs $\mathcal {D}_{i}$, that is, the code of each such $\mathcal {D}_{i}$ is smaller or equal than *n*. By our assumptions on sequence coding (see [33, Section 2.2]), bounds for (codes of) sequences and their concatenations are given by

$$\begin{array}{@{}rcl@{}} \underbrace{\langle k,\ldots,k\rangle}_{m\text{-times}}\leq (k+1)^{2^{m}}; s_{0}^{\smallfrown} s_{1}\leq (s_{0}+s_{1})^{2^{2\mathsf{lh}(s_{0})}}. \end{array} $$

where *lh*(⋅) is the elementary function that outputs the number of the elements of a sequence.

We define an elementary predicate *V*_*n*_(*x*) stating that *x* is proved in predicate logic with a proof whose code is less than *n*:

$$\mathsf{V}_{\!n}(x)\;:\leftrightarrow\;(\exists y\leqslant n)\,(\mathsf{Prf}_{\varnothing}(y,x)) $$

Here $\mathsf {Prf}_{\varnothing }(\cdot ,\cdot )$is elementary and expresses Hilbert-style provability in$\mathsf { PL}(\mathcal {L}^{+})$, predicate logicin the language $\mathcal {L}^{+}$. This *n* is fixed and will be kept so throughout the proof.

We specify the translation $\mathfrak {b}$:it is important to notice that here we are *not* employing the recursion theorem.

$$\begin{array}{@{}rcl@{}} &&(s=t)^{\mathfrak{b}}:=\;s=t (\mathsf{V} x)^{\mathfrak{b}}:=\mathsf{V}_{\!n}(x)\\ &&\cdot^{\mathfrak{b}}\, \text{commutes with prop. connectives} (\forall x\varphi)^{\mathfrak{b}}:=\forall x \varphi^{\mathfrak{b}} \end{array} $$

To verify that $\mathfrak {b}$ is an $\mathcal {L}$-embedding,we check that (VP_1_)and (VD_1_)hold modulo the translation. More generally, we show by induction on the length of the derivation in *B* that,for all $\mathcal {L}^{+}$-sentences *φ*,

$$(8)~~~~~~~~~~~~~~~~~~~~~~~~~~~~~~~~~~~~~~~~~~~~~~~\text{if }B\vdash \varphi, \text{ then } T\vdash \varphi^{\mathfrak{b}}{\kern10pc} $$

It is clear that we obtain (8) when *φ* is a logical or arithmetical axiom of *B*. Let *φ* be of the form$\mathsf {V}(\ulcorner \psi \urcorner )\rightarrow \psi $. Obviously wehave

$$\text{either }\mathbb{N}\models \mathsf{V}\!_{n}(\ulcorner \psi\urcorner)\text{ or }\mathbb{N}\models \neg \mathsf{V}\!_{n}(\ulcorner \psi\urcorner). $$

If the latter, then we are done by Lemma 3 because *V*_*n*_iselementary. If the former disjunct obtains, there is a purely logical derivation$\mathcal {D}_{j}$of *ψ* such that$|\mathcal {D}_{j}|\leq n$. Then there is a purelylogical derivation $\mathcal {D}^{\mathfrak {b}}_{j}$of $\psi ^{\mathfrak {b}}$obtained bytranslating its elements.^14^

For the induction step, we only need to worry about (VP_1_). Now if *φ* is obtained by anapplication of (VP_1_),it has the form $\mathsf {V} (\ulcorner \psi \urcorner )$for *ψ* an$\mathcal {L}^{+}$-sentence and there isa purely logical proof $\mathcal {D}_{i}$of *ψ*. Byassumption, $\,|\mathcal {D}_{i}|\,\leq n$,therefore $T\vdash \mathsf {V}_{\!n}(\ulcorner \psi \urcorner )$byLemma 3. □

Now Proposition 2 immediately yields, besides the consistency of *T*^*V*^ that wasn’t seriously doubted, the conservativity of *T*^*V*^ over *T* for *any* *T* extending **EA**.

#### **Corollary 1**

*T*^*V*^*is a conservative extension of* T.

#### *Proof*

If ${T^{\mathsf {V}}} \vdash \varphi $ and $\varphi \in \mathcal {L}$, then already a finite subsystem $B\subset {T^{\mathsf {V}}}$ proves *φ*. By Proposition 2, $T\vdash \varphi ^{\mathfrak {b}}$. But $\varphi ^{\mathfrak {b}}$ is nothing more than *φ* itself by definition of $\mathfrak {b}$. □

The conservativity of *T*^*V*^ over *T* immediately yields the consistency of *T*^*V*^, relative to the consistency of *T*, that was assumed in Definition 2. It should be noted that the conservativity of $T^{\mathsf {V}}\!\!\upharpoonright $ can be obtained in a straightforward way since any model $\mathcal {M}$ of $T\supseteq \mathsf {EA}$ can be expanded to a model $(\mathcal {M},S)$ of $T^{\mathsf {V}}\!\!\upharpoonright $ where *S* is the set specified in the inductive definition (6). It is not clear to us whether this strategy can be adapted to the full *T*^*V*^. The strategy employed in Proposition 2, however, has the additional advantage of being formalizable with only weak arithmetical assumptions.

Moreover, we obtain another proof of the interpretability of *T*^*V*^ in *T*, for *T* reflexive, by Orey’s compactness theorem:

#### **Corollary 2**

*For* $T\supseteq \mathsf {EA}$ *and reflexive*, *T*^*V*^*is interpretable in* T.

As far as the authors know, the question of the global interpretability of *T*^*V*^ for arbitrary $T\supseteq \mathsf {EA}$ is still open.

### Extending Logical Consequence

It seems natural to wonder whether the reduction methods considered in the previous section can be tweaked to satisfy more principles for *V*. As noticed already by Ketland, the $\mathcal {L}$-embedding $\mathfrak {a}$, without essential modifications, gives us a more substantial theory of logical consequence over reflexive theories encompassing principles such as the following ones:

$$\textup{(\textsf{K})}~~~~~~~~~~~~~~~~~~~~~~~~\mathsf{V} (\ulcorner \varphi\rightarrow\psi\urcorner)\land \mathsf{V} (\ulcorner \varphi\urcorner)\rightarrow \mathsf{V} (\ulcorner \psi\urcorner){\kern10pc} $$

$$(9)~~~~~~~~~~~~~~~~~~~~~~~~~ \neg \mathsf{V} (\ulcorner \mathsf{V}(\ulcorner \varphi\urcorner)\urcorner){\kern17pc} $$

The consistency of the theory *T*^*V*^ + K + 9 is guaranteed by the following corollary to Lemma 2:

#### **Observation 1**

*The theory* *T*^*V*^ + K+*9is* $\mathcal {L}$ *-embeddablein and conservative over* T *for reflexive* $T\supseteq \mathsf {EA}$.

Let’s abbreviate *T*^*V*^ + K by writing *T*^*V*^^+^. Can we obtain analogues of Proposition 2 and Corollary 1 for *T*^*V*^^+^ over arbitrary $T\supseteq \mathsf {EA}$? It turns out that we can, by suitably tweaking the proofs given above.^15^ The fundamental idea is to modify the bound given in the definition of *V*_*n*_ in Proposition 2 to allow for the concatenation of the logical proofs of formulas *φ* and $\varphi \rightarrow \psi $ of $\mathcal {L}^+$ when the translations of the antecedent of K are assumed.

#### **Proposition 3**

*T*^*V*^^+^*islocally* $\mathcal {L}$ *-embeddablein* T.

#### *Proof*

As before, let *B* be a finite subsystem of *T*^*V*^^+^. Again, we fix a standard *n* as bound for the codes of the finitely many logical proofs $\mathcal {D}_{i}$ preceding an application of (VP_1_).

*Let* *C*_*n*_(*x*)*be equivalent to:*

$$\begin{array}{@{}rcl@{}} &&(\exists y\!\leq\! H(n))\;(\mathsf{Prf}_{\varnothing}(y,x)\land (\forall i\leq \mathsf{lh}(y))(\mathsf{det}((y)_{i},y)\rightarrow \mathsf{tru}(y,i)\leq n\land(\exists w\leq n)\\ &&\quad\quad\quad\quad\quad\quad(\mathsf{ Prf}_{\varnothing}(w,(y)_{i})))) \end{array} $$

*where*

$$H(n)=\underbrace{\langle n,\ldots,n\rangle}_{n^{2}\text{-times}}\,^{\smallfrown} \,\underbrace{\langle n,\ldots,n\rangle}_{n^{2}\text{-times}}=\left(2(n+1)^{2^{n^{2}}}\right)^{2^{2n^{2}}} $$

*det*(*x*, *y*)is anelementary predicate expressing that *x* is an ‘only detachable’ member of *y*, that is the proofonly ‘cuts’ *x* via modus ponens and *x* is not a proper subformula of any other member of*y*; *tru*(*x*, *y*)is anelementary function that takes the initial subsequence of *x* with *y* components and outputsits code.^16^Intuitively, *C*_*n*_(*x*)expresses that *x* has a proof in pure logic that (i) applies modus ponens toassumptions that are themselves logically provable with proofs smaller than *n* and (ii) in which all subproofs of these assumptions are also smaller than*n*.

As before, we define the translation$\mathfrak {c}$that, like$\mathfrak {b}$, only replaces outeroccurrences of *V* in proofs,clearly this time with *C*_*n*_and not *V*_*n*_.

The proof now proceeds along similar lines as the proof of Proposition 2 except,of course, for the case of K. In particular, we want to show, for an arbitrary$\varphi \in \mathcal {L}^+$,

$$(10)~~~~~~~~~~~~~~~~~~~~~~~~~~~~~~~~~~~ T\vdash \mathsf{C}_{n}(\ulcorner \varphi\urcorner)\land \mathsf{C}_{n}(\ulcorner \varphi\rightarrow \psi\urcorner)\rightarrow \mathsf{C}_{n}(\ulcorner \psi\urcorner){\kern15pc} $$

As before, if one of $\mathsf {C}_{n}(\ulcorner \varphi \urcorner )$and$\mathsf {C}_{n}(\ulcorner \varphi \rightarrow \psi \urcorner )$are nottrue-in-$\mathbb {N}$,we obtain the claim by Lemma 3. If they are both true, then there are proofs$\mathcal {D}_{0}$and$\mathcal {D}_{1}$in$\mathsf {PL}(\mathcal {L}^{+})$of *φ* and$\varphi \rightarrow \psi $ respecting the conditions above. Since the codes of the detachable members of both proofs willbe smaller than *n*, and so is the number of their subformulas, we can safely assume that

$$|\mathcal{D}_{i}|\leq \langle\underbrace{n,\ldots,n}_{n^{2}\text{-times}}\rangle $$

for *i* ∈{0,1}and that thereis a proof of *ψ* in$\mathsf {PL}(\mathcal {L}^+)$with Gödelnumber ≤ *H*(*n*).

Therefore $\mathbb {N}\vDash \mathsf {C}_{n}(\ulcorner \psi \urcorner )$and $T\vdash \mathsf {C}_{n}(\ulcorner \psi \urcorner )$byLemma 3. □

By the same argument given in Corollary 1:

#### **Corollary 3**

*T*^*V*^^+^*is a conservative extension of T*.

Again, this guarantees the consistency of *T*^*V*^^+^ relative to the consistency of *T*. As above, Orey’s compactness theorem gives us:

#### **Corollary 4**

*For T* *reflexive,* *T*^*V*^^+^*is interpretable in T*.

The results just presented improve the picture discussed in [7, 15] and tell us that in many respects – especially if one focuses on conservativity – primitive logical validity is uniformly reducible to the resources of the base theory for a much wider class of theories than the one considered before. However, we were not able to show the interpretability, let alone the $\mathcal {L}$-embedding, of *T*^*V*^ and *T*^*V*^^+^ in *T*. This is, however, not an unexpected difficulty: by Orey’s and analogous results, there is no gap between local and global interpretability in the context of reflexive theories such as *P***A**. In the case of finitely axiomatizable theories, by contrast, the relationships between these two notions vary considerably and are usually hard to characterize.^17^

From the point of view of the theory of logical validity the lesson to learn is apparent: the combination of (*VP*) and (*VD*)*cannot* be taken to characterize logical validity, which is unparadoxical and uniformly conservative over base theories that contain just a minimum amount of syntactic reasoning.

### Arithmetical Consequence and Hierarchies

In the previous two subsections we analyzed primitive logical consequence based on an introduction rule (VP_1_) for the primitive validity predicate restricted to purely logical proofs. It turns out that no incisions on classical reasoning are needed even if one liberalizes (VP_1_) to arithmetical consequence. We now show that by iterating this idea to the transfinite we obtain a symmetry between the hierarchy of validity predicates suggested in [1, 9] and the hierarchy of local reflection principles for a starting theory *T*. For the sake of determinateness, we assume our starting theory to be **EA**, although the arguments would proceed in an analogous way for any $T\supseteq \mathsf {EA}$.

To define a hierarchy of primitive notions of validity, we assume a notation $(\mathsf {OR},\prec )$ for ordinals up to Γ_0_, available in **EA**,^18^ and a countable stock of predicates *V*_*a*_(*x*) – where *a* ranges over codes of ordinals *α* < Γ_0_, that is we take a Latin alphabet letter to code the corresponding ordinal in the Greek alphabet. We let $\mathcal {L}_{0}$ to be $\mathcal {L}$ itself and $\mathcal {L}_{\alpha +1}$ is $\mathcal {L}_{\alpha } \cup \{\mathsf {V}_{\!a}\}$; $\mathcal {L}_{\lambda }$, for *λ* limit, contains all *V*_*b*_ for *β* < *λ*.

#### **Definition 3** (Hierarchical validity)

*Let* **S**^0^ := **EA**. *For successor ordinals*, *with* *α* < Γ_0_, **S**^*α*+1^*in* $\mathcal {L}_{\alpha }$ *isdefined as follows*: 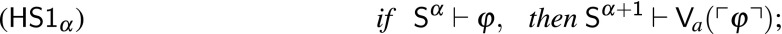

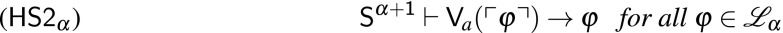

$$\textup{(HL)}~~~~~~~~~~~~~~~~~~~~~~~~~~~~~~~~~~~~~~~~~~~~~~~~~~~~~~~~~~~\bigcup_{\beta<\lambda} \mathsf{S}^{\beta}\;\;\text{ for}\, \lambda\, \text{limit.}{\kern17pc} $$

We briefly comment on the halting point Γ_0_: it is motivated by the availability of natural notation systems and corresponding well-ordering proofs in the theories **S**^*α*^. Variations are obviously possible: notations for more ordinals are possible in **EA**, although the details will bring us too far from our main concerns here. By contrast, if one wants to stick with ordinals that are provably well-ordered in **EA**, one would need to stop at *ω*^3^. We claim that this hierarchy of validity predicates is closely related to the following, well-known hierarchy of local reflection principles over **EA**, again for ordinals *α*, *λ* < Γ_0_:

$$\begin{array}{@{}rcl@{}} \mathsf{R}^{0}:= \mathsf{EA}; \quad\qquad\quad \mathsf{R}^{\alpha+1}:=\mathsf{R}^{\alpha}+ \mathsf{Rfn}(\mathsf{R}^{\alpha}); \quad\qquad\quad \mathsf{ R}^{\lambda} := \bigcup\limits_{\beta<\lambda} \mathsf{R}^{\beta}. \end{array} $$

where

$$\mathsf{Rfn}(T):=\mathsf{Pr}_{T}(\ulcorner \varphi\urcorner)\rightarrow \varphi \;\;\text{ for all }\varphi\in \mathcal{L}_{T}. $$

The formalization of provability for the theories *R*^*α*^ can be carried out in a standard way once a notation for the ordinals and suitable well-ordering proofs are available.

#### **Proposition 4**

*For* *α* < Γ_0_, **S**^*α*^*is* $\mathcal {L}$ *-embeddablein* *R*^*α*^.

#### *Proof*

For each *α* < Γ_0_, we define a translation $\mathfrak d\colon \mathcal {L}_{<\alpha }\to \mathcal {L}$ as follows, for all *β* < *α*, and where $\mathcal {L}_{<\alpha }:=\bigcup _{\beta <\alpha }\mathcal {L}_{\beta }$:

$$\begin{array}{@{}rcl@{}} &&(s=t)^{\mathfrak d}:= (s=t) (\neg\varphi)^{\mathfrak d}:=\neg \varphi^{\mathfrak d}\\ &&(\varphi\land \psi)^{\mathfrak d}:= \varphi^{\mathfrak d}\land \psi^{\mathfrak d} (\forall x\varphi)^{\mathfrak d}:=\forall x\varphi^{\mathfrak d}\\ &&(\mathsf{V}_{\! b}(x))^{\mathfrak d}:= \mathsf{Pr}_{\mathsf{R}^{\beta}}({\mathfrak d}(x)) \end{array} $$

Now we argue inductively given that *V*^0^ = *R*^0^ and that limit stages are not problematic. For *H***S**2_*α*_, if, in *R*^*α*+1^, we have$\mathsf {Pr}_{\mathsf {R}^{\alpha }}(\mathfrak {d}(\ulcorner \varphi \urcorner ))$ for a standard *φ*, we can conclude $\varphi ^{\mathfrak d}$ by applying the reflection principle of level *α* since$\mathsf {Sent}_{\mathcal {L}}(\mathfrak {d}\ulcorner \varphi \urcorner )$, provably in **EA**. For *H***S**1_*α*_, we can safely assume that $\mathsf {R}^{\alpha }\vdash \varphi ^{\mathfrak {d}}$. Therefore already in **EA**, $\mathsf {Pr}_{\mathsf {R}^{\alpha }}({\mathfrak {d}}(\ulcorner \varphi \urcorner ))$. □

Proposition 4 suggests at least the following two remarks: for the reader interested in the mathematical strength of the theories **S**^*α*^, by a result of Beklemishev [2], these theories will prove no more Π_1_-arithmetical sentences than *ω*^*α*^ iterated consistency progressions over **EA**. At the philosophical level, the theories **S**^*α*^ embody a notion of arithmetical validity corresponding to a proper extension of arithmetical provability in the starting theory **EA** stratified along ordinal paths that are meaningful in the starting theory. No incision on classical logic is needed at this stage. However, as pointed out also in [9], the formulation of the theories **S**^*α*^ relies on how many ordinals we can code in the starting theory. In order to read off a notion of validity from this stratified picture there seem to be only two options: either validity is inherently stratified, or there is a specific countable ordinal *α* such that **S**^*α*^ fulfills the requirements we are willing to ascribe to the notion of validity. Neither of these alternatives, however, is completely satisfactory; for one thing, there is no reason to think that validity should be stratified, unless one is happy to concede that also truth is a stratified notion. Moreover, the countable ordinals that are provably well-founded in arithmetical theories vary considerably, and it is highly implausible that the notion of validity should be tied to these implementation details.

This is not to say, however, that stratified validity lacks importance. Even if it doesn’t afford a viable notion of validity, it gives us a tool to generate validities starting from valid inferences in the base language. This picture will be improved in the in the next two sections, where we will turn the hierarchical increase of validities into a positive inductive definition. This technical shift will yield a truly self-applicable notion of validity whose extension and properties are independent of how we represent ordinals. As in the case of truth, this will require restricting classical reasoning.

## A New Construction for Naïve Validity

In this section we propose a way of transcending the stratified picture of validity that generalizes Kripke’s method to provide models for languages with a self-applicable truth predicate (see [16]). We will be mainly concerned with providing a class of models that makes the naïve principles for validity consistent, and not so much with formulating effectively presented theories of validity (as in the previous section).^19^ The models are obtained via fixed points of an inductive construction. Similarly to what happens in Kripke’s theory of truth, we have only one validity predicate that can be introduced without restrictions, via (*VP*). At a fixed point, (*VP*) and all the principles that are accepted in the construction can be iterated arbitrarily, thus internalizing *all* the inferences deriving from validities of the base language.

### Initial Sequents and Rules of Inferences

Since, in the perspective of an unstratified picture of validity, (*VP*) is not in question, what are we do to with the V-Curry Paradox? We have seen in the introduction that the paradox forces a restriction of contraction, cut, or (*VD*). The idea of transcending the hierarchical conception of validity via an inductive definition is at odds with (*VD*), even though it calls for an unrestricted acceptance of contraction and cut.^20^ For sure, (*VD*) looks perfectly fine if we read Val as an unspecified ‘following from’ and clearly also as the notion of logical validity studied in Sections 2.2 and 2.3, but things are different if we accept (*VP*) unrestrictedly: in so doing, we take Val-statements to represent a naïve notion of consequence, namely meta-inferences that hold in virtue of logical, base-theoretic, and validity-theoretic principles. However, in the presence of full (*VD*), this idea translates into the acceptance of sentences that we might not want to accept, such as *ν*.

In the perspective of transcending the hierarchy of validity predicates, one might think that the problem with (*VD*) is that it allows us to conclude *ψ* on the *assumption* that *φ* and $\mathsf {Val}(\ulcorner \varphi \urcorner , \ulcorner \psi \urcorner )$ hold. However, if the validity predicate represents meta-theoretical inferences (possibly nested, due to its iterability), one might want to employ an elimination rule that is based on Val-statements that are actually *accepted*, rather than arbitrarily assumed. The following elimination rule for Val embodies this intuition: 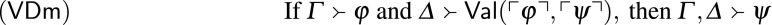


Adopting (*VDm*), however, is not sufficient to avoid the V-Curry Paradox in the presence of *reflexivity*: 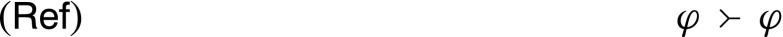 In fact, (*Ref*) and (*VDm*) together immediately yield (*VD*). So, a proof of ⊥ is now easy to obtain via a modification of the V-Curry derivation given in the introduction, using (*Ref*) and (*VDm*).

How can we avoid this new path to triviality? Our proposed solution consists in the development of a Kripke-style positive inductive definition that, while restricting (*VD*) and (*Ref*), consistently satisfies (*VP*), (*VDm*), contraction, cut, and indeed every other classically valid rule of inference (with nonzero premises).^21^ More generally, our construction will operate uniform restrictions on initial sequents: this harmonizes with the motivation to restrict (*VD*) outlined above, since arbitrary initial sequents might contain Val-sentences codifing problematic inferences. Rules of inference, by contrast, are safe: if we can control the sequents that we accept, we can adopt all such rules. A Kripke-style construction along the lines of the one developed here has been hinted at by Field in [9]. Meadows [20] also develops an inductive construction that recovers all Beall and Murzi’s principles. His construction also rejects reflexivity but, unlike ours, it is not closed under contraction and cut.^22^

### The KV-Construction

The generalization of Kripke’s construction we propose here consists in dealing with *sequents* rather than single sentences. By ‘sequent’, from now on, we will mean an object of the form ${\Gamma } \Rightarrow {\Delta }$, where Γ (the antecedent) and Δ (the consequent) are finite sets of $\mathcal {L}_{V}$-sentences.

Let us describe our approach informally. The following definitions formalize the intuition outlined above, by enabling us to:

- start with a set of sequents containing at least the ones the form ${\Gamma } \Rightarrow {\Delta }, s_{0}=t_{0}$ and $s_{1}=t_{1}, {\Gamma } \Rightarrow {\Delta }$, for *s*_0_ = *t*_0_ an atomic arithmetical truth, and *s*_1_ = *t*_1_ an atomic arithmetical falsity,
- apply the operational and structural rules of inference to them,
- internalize the sequents so obtained within the validity predicate, interpreting Val via principles that are modelled after the rules of inference for the classical material conditional $\supset $.

This process can be iterated: we apply again the operational, structural, and Val rules, and so on *ad infinitum*. At some ordinal stage, this process reaches a fixed point, which provides the desired interpretation of Val.

#### **Definition 4**

*Let* $S \subseteq \omega $, *anddefine the set* *S*^+^*as follows.* *n* ∈ *S*^+^*if*:

(i) *n* ∈ *S*; *or*
(ii) *n is* ${\Gamma } \Rightarrow s=t, {\Delta }$, *and* $\mathbb {N} \models s=t$; *or*
(iii) *n is* ${\Gamma }, s=t \Rightarrow {\Delta }$, *and* $\mathbb {N} \models s\neq t$; *or*
(iv) *n is* ${\Gamma } \Rightarrow \varphi \wedge \psi , {\Delta }$, *and* ${\Gamma } \Rightarrow \varphi , {\Delta } \in S$, *and* ${\Gamma } \Rightarrow \psi , {\Delta } \in S$; *or*
(v) *n is* ${\Gamma }, \varphi \wedge \psi \Rightarrow {\Delta }$, *and* ${\Gamma }, \varphi , \psi \Rightarrow {\Delta } \in S$; *or*
(vi) *n is* ${\Gamma } \Rightarrow \varphi \vee \psi , {\Delta }$, *and* ${\Gamma } \Rightarrow \varphi , \psi , {\Delta } \in S$; *or*
(vii) *n is* ${\Gamma }, \varphi \vee \psi \Rightarrow {\Delta }$, *and* ${\Gamma }, \varphi \Rightarrow {\Delta } \in S$, *and* ${\Gamma }, \psi \Rightarrow {\Delta } \in S$; *or*
(viii) *n is* ${\Gamma } \Rightarrow \forall x \varphi (x), {\Delta }$, *and for all* $t \in \mathsf { Cter_{\mathcal {L}_{V}}}$, ${\Gamma } \Rightarrow \varphi (t), {\Delta } \in S$; *or*
(ix) *n is* ${\Gamma }, \forall x \varphi (x) \Rightarrow {\Delta }$, *and for some* $t \in \mathsf { Cter_{\mathcal {L}_{V}}}$, ${\Gamma }, \varphi (t) \Rightarrow {\Delta } \in S$; *or*
(x) *n is* ${\Gamma } \Rightarrow \mathsf {Val}(\ulcorner \varphi \urcorner , \ulcorner \psi \urcorner ), {\Delta }$, *and* ${\Gamma }, \varphi \Rightarrow \psi , {\Delta } \in S$; *or*
(xi) *n is* ${\Gamma }, \mathsf {Val}(\ulcorner \varphi \urcorner , \ulcorner \psi \urcorner ) \Rightarrow {\Delta }$, *and* ${\Gamma } \Rightarrow \varphi , {\Delta } \in S$, *and* ${\Gamma }, \psi \Rightarrow {\Delta } \in S$.

$\mathsf {Cter_{\mathcal {L}_{V}}}$ is an elementary predicate representing the set of (codes of) closed terms of $\mathcal {L}_{V}$. Let *ζ*(*n*, *S*) abbreviate items (i)-(xi). We can express this definition with an operator ${\Psi } : \mathcal {P}(\omega ) \longmapsto \mathcal {P}(\omega )$ defined as Ψ(*S*) := {*n* ∈ *ω* | *ζ*(*n*, *S*)}. The operator Ψ is *increasing* and *monotone*, namely:

- for every $S \subseteq \omega $, we have that $S \subseteq {\Psi }(S)$;
- for every $S_{1}, S_{2} \subseteq \omega $, if $S_{1} \subseteq S_{2}$, then ${\Psi }(S_{1}) \subseteq {\Psi }(S_{2})$.

For every $S\subseteq \omega $, the set

$$S_{\Psi} := \bigcup_{\alpha \in \textit{Ord}} {\Psi}^{\alpha}(S)$$

is a *fixed point* of Ψ, since Ψ(*S*_Ψ_) = *S*_Ψ_. *S*_Ψ_ is said to be the fixed point of Ψ*generated byS*. Let’s denote with *I*_Ψ_ the fixed point of Ψ generated by the empty set:

$$\mathsf{I}_{\Psi} := \bigcup_{\alpha \in \textit{Ord}}{\Psi}^{\alpha}(\varnothing). $$

*I*_Ψ_ is the *least fixed point* of Ψ: for every $S \subseteq \omega $, $\mathsf {I}_{\Psi } \subseteq S_{\Psi }$.

In the next section, we prove that *I*_Ψ_ can be used to interpret $\mathcal {L}_{V}$-sequents in a non-trivial way. We will then investigate the behaviour of the structural rules and of the validity-theoretical principles in fixed points of Ψ.

## Main Properties of the KV-Construction

We start by showing that there are consistent fixed points of Ψ. A fixed point *S*_Ψ_ is *consistent* if it does not contain the empty sequent $\varnothing \Rightarrow \varnothing $. Consistency typically avoids triviality: if $\varnothing \Rightarrow \varnothing $ is in a fixed point closed under weakening, then *every* sequent is in that fixed point.^23^

### **Proposition 5**

*I*_Ψ_ *is consistent.*

### *Proof*

The proof is by induction on the stages $\mathsf {I}^{\alpha }_{\Psi }$ *of the construction of* *I*_Ψ_. The claim is trivial for $\mathsf {I}^{0}_{\Psi }$ and $\mathsf {I}^{1}_{\Psi }$. Assuming the claim for the stage $\mathsf { I}^{\alpha }_{\Psi }$, one simply notices that the stage $\mathsf {I}^{\alpha +1}_{\Psi }$ is obtained from $\mathsf {I}^{\alpha }_{\Psi }$ by adding to it all sequents resulting from an application of the clauses of Definition 4. It is then clear that, if $\varnothing \Rightarrow \varnothing $ is not in $\mathsf {I}^{\alpha }_{\Psi }$, no such clause can introduce it in $\mathsf {I}^{\alpha +1}_{\Psi }$. The limit case follows straightforwardly from the successor cases. □

We first notice that every stage $\mathsf {I}_{\Psi }^{\alpha }$ of *I*_Ψ_ is closed under left and right weakening. By construction, any sequent ${\Gamma } \Rightarrow {\Delta }$ in $\mathsf {I}_{\Psi }^{\alpha }$ is obtained by applying a series of Ψ-clauses to sequents containing arithmetical truths or falsities, with arbitrary side sentences. Therefore, in order to have ${\Gamma }, {\Gamma }^{\prime } \Rightarrow {\Delta },{\Delta }^{\prime }$ in $\mathsf { I}_{\Psi }^{\alpha }$, we simply consider the same succession of Ψ-clauses applied to starting sequents with ${\Gamma }^{\prime }$ and ${\Delta }^{\prime }$ as extra side sentences.

### **Lemma 4** (Weakening)

*For every ordinal* *α*, *if* ${\Gamma } \Rightarrow {\Delta }$ *is in* $\mathsf {I}_{\Psi }^{\alpha }$, *then for every* ${\Gamma }^{\prime }, {\Delta }^{\prime } \subseteq \mathsf {Sent_{\mathcal {L}_{V}}}$, *the sequent* ${\Gamma }, {\Gamma }^{\prime } \Rightarrow {\Delta },{\Delta }^{\prime }$ *is in* $\mathsf { I}_{\Psi }^{\alpha }$.

Also, since we are dealing with finite sets, left and right contraction hold for every sequent in every stage of the construction of *I*_Ψ_.

### **Lemma 5** (Contraction)

*For every ordinal* *α*, *if* ${\Gamma },\varphi ,\varphi \Rightarrow {\Delta }$ *is in* $\mathsf {I}_{\Psi }^{\alpha }$, *then* ${\Gamma },\varphi \Rightarrow {\Delta }$ *is in* $\mathsf {I}_{\Psi }^{\alpha }$. *Similarly*, *if* ${\Gamma }\Rightarrow \psi ,\psi ,{\Delta }$ *is in* $\mathsf { I}_{\Psi }^{\alpha }$, *then* ${\Gamma }\Rightarrow \psi ,{\Delta }$ *is in* $\mathsf {I}_{\Psi }^{\alpha }$.

A crucial feature of our construction is that, at any stage, sequents are *grounded* in at least one sentence in their antecedent or consequent.

### **Lemma 6** (Groundedness)

*For every ordinal* *αand every sequent* ${\Gamma } \Rightarrow {\Delta }$, *if* ${\Gamma } \Rightarrow {\Delta }$ *is in* $\mathsf {I}_{\Psi }^{\alpha }$, *then there is at least one sentence* *φin* Γ*such that* $\varphi \Rightarrow \varnothing $ *is in* $\mathsf {I}_{\Psi }^{\alpha }$, *or at least one sentence* *ψin* Δ*such that* $\varnothing \Rightarrow \psi $ *is in* $\mathsf {I}_{\Psi }^{\alpha }$.

### *Proof*

We reason by induction on the construction of *I*_Ψ_. The claim is trivial for $\mathsf {I}_{\Psi }^{0}$. For $\mathsf {I}_{\Psi }^{1}$, the claim is also immediate since this set contains only sequents with atomic arithmetical truths in the consequent or atomic arithmetical falsities in the antecedent. We now assume the claim up to *α*, and prove it for *α* + 1. Let ${\Gamma } \Rightarrow {\Delta } \in \mathsf {I}_{\Psi }^{\alpha +1}$ be obtained by applying one Ψ -clause to sequents in $\mathsf {I}_{\Psi }^{\alpha }$. We consider two cases.

We first deal with the Ψ-clause for introducing ∀ on the right: in this case ${\Gamma } \Rightarrow {\Delta }$ has the form ${\Gamma } \Rightarrow {\Delta }^{\prime },\forall x \varphi (x)$. The sequents in $\mathsf {I}_{\Psi }^{\alpha }$ from which ${\Gamma } \Rightarrow {\Delta }^{\prime }, \forall x \varphi (x)$ is obtained, then, have the following form:

$$(11) ~~~~~~~~~~~~~~~~~~~~~~~~~~~~~~~~~~~~~{\Gamma} \Rightarrow {\Delta}^{\prime}, \varphi(t_{0}), \ldots,\, {\Gamma} \Rightarrow {\Delta}^{\prime}, \varphi(t_{n}), \ldots{\kern14pc} $$

By induction hypothesis, for every ${\Gamma } \Rightarrow {\Delta }^{\prime }, \varphi (t_{i})$ in (4), there is a *ψ*_*i*_in Γ sucht hat $\psi _{i} \Rightarrow \varnothing $ belongs to $\mathsf {I}_{\Psi }^{\alpha }$, or a *χ*_*i*_in${\Delta }^{\prime }, \varphi (t_{i})$ such that $\varnothing \Rightarrow \chi _{i}$ belongs to $\mathsf {I}_{\Psi }^{\alpha }$. If, forsome *i*, *ψ*_*i*_or *χ*_*i*_are in Γ or${\Delta }^{\prime }$, we are done. If there is no *i* such that *ψ*_*i*_ or *χ*_*i*_ are in Γ or${\Delta }^{\prime }$, the induction hypothesis gives us that $\varnothing \Rightarrow \varphi (t_{i})$ is in $\mathsf {I}_{\Psi }^{\alpha }$for all *i*. Therefore, an application of the Ψ-clause(ix) gives us that $\varnothing \Rightarrow \forall x \varphi (x)$ is in $\mathsf {I}_{\Psi }^{\alpha +1}$,as desired.

If ${\Gamma } \Rightarrow {\Delta }$is obtained via the Ψ-clause (xi) of Definition 4,then it has the form ${\Gamma }^{\prime }, \mathsf {Val}(\ulcorner \varphi _{0}\urcorner ,\ulcorner \varphi _{1}\urcorner )\Rightarrow {\Delta }$ and $\mathsf {I}_{\Psi }^{\alpha }$ contains ${\Gamma }^{\prime } \Rightarrow \varphi _{0},{\Delta }$ and ${\Gamma }^{\prime },\varphi _{1}\Rightarrow {\Delta }$. If the induction hypothesis gives us sequents $\psi \Rightarrow \varnothing $or $\varnothing \Rightarrow \chi $ where *ψ* or *χ* are in ${\Gamma }^{\prime }$or Δ respectively, we are done. In the only other case, the induction hypothesis gives us that$\varphi _{1}\Rightarrow \varnothing $ and $\varnothing \Rightarrow \varphi _{0}$ are in $\mathsf {I}_{\Psi }^{\alpha }$. By the Ψ-clause (xi) of Definition 4, then, we get $\mathsf {Val}(\ulcorner \varphi _{0}\urcorner ,\ulcorner \varphi _{1}\urcorner )\Rightarrow \varnothing $in $\mathsf {I}_{\Psi }^{\alpha +1}$. □

To prove the closure of the stages of the construction of *I*_Ψ_ under cut, we need the following inversion lemma.

### **Lemma 7** (Inversion)

*For every ordinal* *α*, *the following holds*:

(i) *If* ${\Gamma } \Rightarrow \varphi \wedge \psi , {\Delta }$ *is in* $\mathsf {I}_{\Psi }^{\alpha }$, *then* ${\Gamma } \Rightarrow \varphi , {\Delta }$ *is in* $\mathsf {I}_{\Psi }^{\alpha }$ *and* ${\Gamma } \Rightarrow \psi , {\Delta } $ *is in* $\mathsf { I}_{\Psi }^{\alpha }$.
(ii) *If* ${\Gamma }, \varphi \wedge \psi \Rightarrow {\Delta }$ *is in* $ \mathsf {I}_{\Psi }^{\alpha }$, *then* ${\Gamma }, \varphi , \psi \Rightarrow {\Delta }$ *is in* $\mathsf {I}_{\Psi }^{\alpha }$.
(iii) *If* ${\Gamma } \Rightarrow \varphi \vee \psi , {\Delta }$ *is in* $ \mathsf {I}_{\Psi }^{\alpha }$, *then* ${\Gamma } \Rightarrow \varphi , \psi , {\Delta }$ *is in* $ \mathsf {I}_{\Psi }^{\alpha }$.
(iv) *If* ${\Gamma }, \varphi \vee \psi \Rightarrow {\Delta }$ *is in* $ \mathsf {I}_{\Psi }^{\alpha }$, *then* ${\Gamma }, \varphi \Rightarrow {\Delta }$ *is in* $ \mathsf {I}_{\Psi }^{\alpha }$ *and* ${\Gamma }, \psi \Rightarrow {\Delta } $ *is in* $\mathsf { I}_{\Psi }^{\alpha }$.
(v) *If* ${\Gamma } \Rightarrow \forall x \varphi (x), {\Delta }$ *is in* $\mathsf {I}_{\Psi }^{\alpha }$, *then for all* $t \in \mathsf {Cter_{\mathcal {L}_{V}}}$: ${\Gamma } \Rightarrow \varphi (t), {\Delta }$ *is in* $ \mathsf { I}_{\Psi }^{\alpha }$.
(vi) *If* ${\Gamma }, \forall x \varphi (x) \Rightarrow {\Delta } $ *is in* $\mathsf {I}_{\Psi }^{\alpha }$, *then for some* $t \in \mathsf {Cter_{\mathcal {L}_{V}}}$: ${\Gamma }, \varphi (t) \Rightarrow {\Delta }$ *is in* $\mathsf { I}_{\Psi }^{\alpha }$.
(vii) *If* ${\Gamma } \Rightarrow \mathsf {Val}(\ulcorner \varphi \urcorner , \ulcorner \psi \urcorner ), {\Delta }$ *is in* $\mathsf {I}_{\Psi }^{\alpha }$, *then* ${\Gamma }, \varphi \Rightarrow \psi , {\Delta }$ *is in* $\mathsf {I}_{\Psi }^{\alpha }$.
(viii) *If* ${\Gamma }, \mathsf {Val}(\ulcorner \varphi \urcorner , \ulcorner \psi \urcorner ) \Rightarrow {\Delta }$ *is in* $\mathsf {I}_{\Psi }^{\alpha }$, *then* ${\Gamma } \Rightarrow \varphi , {\Delta }$ *is in* $\mathsf {I}_{\Psi }^{\alpha }$ *and* ${\Gamma }, \psi \Rightarrow {\Delta }$ *is in* $\mathsf {I}_{\Psi }^{\alpha }$.

### *Proof*

We will only consider case (viii). Le *α* + 1 be the least ordinal such that ${\Gamma }, \mathsf {Val}(\ulcorner \varphi _{0}\urcorner ,\ulcorner \varphi _{1}\urcorner ) \Rightarrow {\Delta }$ is in $\mathsf {I}_{\Psi }^{\alpha +1}$. Then either (a) ${\Gamma }, \mathsf {Val}(\ulcorner \varphi _{0}\urcorner ,\ulcorner \varphi _{1}\urcorner ) \Rightarrow {\Delta }$ is obtained by the Ψ-clause (xi) of Definition 4 or (b) it is obtained via a different Ψ-clause. In case (a) ${\Gamma }, \varphi _{1} \Rightarrow {\Delta }$ *and* ${\Gamma }\Rightarrow \varphi _{0},{\Delta }$ are in $\mathsf {I}_{\Psi }^{\alpha }$ and we are done. If (b), there are several sub-cases to consider: we just deal with an application of the Ψ-clause (viii), which yields a sequent of the form ${\Gamma }, \mathsf {Val}(\ulcorner \varphi _{0}\urcorner ,\ulcorner \varphi _{1}\urcorner ) \Rightarrow {\Delta }^{\prime },\forall x\varphi (x)$, with ${\Delta }={\Delta }^{\prime },\forall x\varphi (x)$. In this case ${\Gamma }, \mathsf {Val}(\ulcorner \varphi _{0}\urcorner ,\ulcorner \varphi _{1}\urcorner ) \Rightarrow {\Delta }^{\prime },\varphi (t_{i})$ is in $\mathsf {I}_{\Psi }^{\alpha }$ for every *i* for some formula *φ*(*x*). By induction hypothesis we obtain, in $\mathsf {I}_{\Psi }^{\alpha }$, sequents of the form

$$\begin{array}{@{}rcl@{}} &(\star)\hspace{10pt}{\Gamma}, \varphi_{1} \Rightarrow {\Delta}^{\prime},\varphi(t_{i}) \qquad\qquad (\dagger)\hspace{10pt} {\Gamma} \Rightarrow \varphi_{0},{\Delta}^{\prime},\varphi(t_{i}) \end{array} $$

for every *i*. By applying the Ψ-clause (viii) to all sequents of the form (⋆) and (‡) respectively, we obtain that ${\Gamma }, \varphi _{1} \Rightarrow {\Delta }^{\prime },\forall x \varphi (x)$ and ${\Gamma } \Rightarrow \varphi _{0},{\Delta }^{\prime },\forall x \varphi (x)$ in $\mathsf {I}_{\Psi }^{\alpha +1}$, as desired. □

Finally, we show that every stage of the construction of *I*_Ψ_ is closed under cut.

### **Proposition 6** (Closure under cut)

*For every* *α*, *if* ${\Gamma }\Rightarrow {\Delta },\varphi $ *and* $\varphi ,{\Gamma }\Rightarrow {\Delta }$ *are in* $\mathsf {I}_{\Psi }^{\alpha }$, *then also* ${\Gamma }\Rightarrow {\Delta }$ *is in* $\mathsf {I}_{\Psi }^{\alpha }$.

### *Proof*

The proof is by induction. The case for $\mathsf {I}_{\Psi }^{0}$ is trivial. The case for $\mathsf {I}_{\Psi }^{1}$ is also immediately obtained since, for ${\Gamma }\Rightarrow {\Delta },\varphi $ and $\varphi ,{\Gamma }\Rightarrow {\Delta }$ to be in $\mathsf {I}_{\Psi }^{1}$, Γ or Δ have to contain at least one atomic arithmetical falsity or truth respectively.

Let us suppose that, for *α* > 0, ${\Gamma }\Rightarrow {\Delta },\varphi $ and $\varphi ,{\Gamma }\Rightarrow {\Delta }$ are in $\mathsf {I}_{\Psi }^{\alpha +1}$. There are three main cases to be considered: (a) in the first, ${\Gamma }\Rightarrow {\Delta },\varphi $ and $\varphi ,{\Gamma }\Rightarrow {\Delta }$ are obtained by means of a Ψ-clause that introduces *φ*; (b) in the second, only one of ${\Gamma }\Rightarrow {\Delta },\varphi $ and $\varphi ,{\Gamma }\Rightarrow {\Delta }$ is obtained via a Ψ-clause that introduces *φ*; (c) in the third, neither ${\Gamma }\Rightarrow {\Delta },\varphi $ nor $\varphi ,{\Gamma }\Rightarrow {\Delta }$ is obtained via a Ψ-clause that introduces *φ*.

- (a) We consider the case in which *φ* is of the form $\mathsf {Val}(\ulcorner \varphi _{0}\urcorner ,\ulcorner \varphi _{1}\urcorner )$.Therefore the sequents
  $$\begin{array}{@{}rcl@{}} \text{(I)}\hspace{10pt}{\Gamma},\varphi_{0}\Rightarrow\varphi_{1}, {\Delta} \qquad\qquad \text{(II)}\hspace{10pt} {\Gamma}\Rightarrow \varphi_{0},{\Delta} \qquad\qquad \text{(III)}\hspace{10pt} {\Gamma},\varphi_{1}\Rightarrow {\Delta} \end{array} $$
  are in $\mathsf {I}_{\Psi }^{\alpha }$. By the weakening lemma applied to (II), also ${\Gamma }\Rightarrow \varphi _{0},\varphi _{1},{\Delta }$ is in $\mathsf {I}_{\Psi }^{\alpha }$. By induction hypotesis, since (I) is in $\mathsf {I}_{\Psi }^{\alpha }$, also ${\Gamma }\Rightarrow \varphi _{1},{\Delta }$ is in $\mathsf {I}_{\Psi }^{\alpha }$. Therefore, since (III) is also in $\mathsf {I}_{\Psi }^{\alpha }$, ${\Gamma }\Rightarrow {\Delta }$ will be in $\mathsf {I}_{\Psi }^{\alpha }\subseteq \mathsf {I}_{\Psi }^{\alpha +1}$ as well, as desired.
- (b) We only consider the case in which *φ* is $\mathsf {Val}(\ulcorner \varphi _{0}\urcorner ,\ulcorner \varphi _{1}\urcorner )$. We assume, moreover, that ${\Gamma }\Rightarrow {\Delta }, \mathsf {Val}(\ulcorner \varphi _{0}\urcorner ,\ulcorner \varphi _{1}\urcorner )$ is obtained via the Ψ-clause (viii) from sequents in $\mathsf {I}_{\Psi }^{\alpha }$ of the form
  $$\text{(IV)}\hspace{10pt}{\Gamma}\Rightarrow {\Delta}^{\prime}, \varphi(t_{i}), \mathsf{Val}(\ulcorner \varphi_{0}\urcorner,\ulcorner\varphi_{1}\urcorner) $$
  for all *i* ∈ *ω*, and that$\mathsf {Val}(\ulcorner \varphi _{0}\urcorner ,\ulcorner \varphi _{1}\urcorner ),{\Gamma }\Rightarrow {\Delta }$ is obtainedvia the Ψ-clause(xi) from ${\Gamma }\Rightarrow \varphi _{0},{\Delta }$ and ${\Gamma },\varphi _{1}\Rightarrow {\Delta }$ in $\mathsf {I}_{\Psi }^{\alpha }$. On the one hand, by the inversion lemma applied to all sequentsof the form (IV), we obtain that all sequents of the form ${\Gamma },\varphi _{0}\Rightarrow {\Delta }^{\prime }, \varphi (t_{i}),\varphi _{1}$ are in $\mathsf {I}_{\Psi }^{\alpha }$. By the weakening lemma, since ${\Delta }={\Delta }^{\prime },\forall x\varphi (x)$,
  $$\text{(V)}\hspace{10pt}{\Gamma},\varphi_{0}\Rightarrow {\Delta}, \varphi(t_{i}),\varphi_{1} $$
  is in $\mathsf {I}_{\Psi }^{\alpha }$ for all *i* ∈ *ω*. On the other, from the fact that ${\Gamma }\Rightarrow \varphi _{0},{\Delta }$ and ${\Gamma },\varphi _{1}\Rightarrow {\Delta }$ are in $\mathsf {I}_{\Psi }^{\alpha }$, the weakening lemma gives us
  $$\begin{array}{@{}rcl@{}} \text{(VI)}\hspace{10pt}{\Gamma}\Rightarrow\varphi(t_{i}),\varphi_{0}, \varphi_{1},{\Delta} \qquad\qquad \text{(VII)}\hspace{10pt} {\Gamma},\varphi_{1}\Rightarrow\varphi(t_{i}), {\Delta} \end{array} $$
  in $\mathsf {I}_{\Psi }^{\alpha }$. By induction hypotesis, since for all *i* ∈ *ω*(V) and (VI) are in $\mathsf {I}_{\Psi }^{\alpha }$, also ${\Gamma }\Rightarrow \varphi (t_{i}), \varphi _{1},{\Delta }$ is in $\mathsf {I}_{\Psi }^{\alpha }$ for all *i* ∈ *ω*. Therefore, since for all *i* ∈ *ω* (VII) is also in $\mathsf {I}_{\Psi }^{\alpha }$, ${\Gamma }\Rightarrow \varphi (t_{i}), {\Delta }$ will be in $\mathsf {I}_{\Psi }^{\alpha }$ for all *i* ∈ *ω*. An application of the Ψ-clause (viii) gives us the desired result.
- (c) We consider the case in which ${\Gamma }\Rightarrow {\Delta },\varphi $ and $\varphi ,{\Gamma }\Rightarrow {\Delta }$are obtained by application of the Ψ-clause (viii) to sequents in $\mathsf {I}_{\Psi }^{\alpha }$ of the form
  $$\begin{array}{@{}rcl@{}} \text{(VIII)}\hspace{10pt}{\Gamma}\Rightarrow{\Delta}^{\prime},\varphi_{0}(t_{i}),\varphi \qquad\qquad \text{(IX)}\hspace{10pt}\varphi,{\Gamma}\Rightarrow{\Delta}^{\prime\prime},\varphi_{1}(t_{i}) \end{array} $$
  for all *i* ∈ *ω*. Bythe groundedness lemma applied to all sequents of the form (VIII), we obtain, for each *i* ∈ *ω*, that either thereis a sequent $\psi _{i}\Rightarrow \varnothing $ is in $\mathsf {I}_{\Psi }^{\alpha }$ for *ψ*_*i*_ ∈ Γ, or there is a sequent $\varnothing \Rightarrow \chi _{i}$ in $\mathsf {I}_{\Psi }^{\alpha }$ with $\chi _{i}\in {\Delta }^{\prime },\varphi _{0}(t_{i}),\varphi $. In the former case, we are done by the weakening lemma; in the latter case, if$\chi _{i}\in {\Delta }^{\prime }$we are also done by weakening, otherwise we reason as follows. If *χ*_*i*_is *φ*_0_(*t*_*i*_) for all *i*, an application of the Ψ-clause (viii) gives us that $\varnothing \Rightarrow \forall x\varphi _{0}(x)$ is in $\mathsf {I}_{\Psi }^{\alpha +1}$, therefore the claim follows by the weakening lemma. If *χ*_*i*_is *φ* for some *i*, we apply the groundedness lemma to all sequents of the form (IX). By the consistency of *I*_Ψ_, the induction hypothesis cannot give us $\varphi \Rightarrow \varnothing $ in $\mathsf {I}_{\Psi }^{\alpha }$. In all other possible outcomes of the groundedness lemma applied to all sequents of the form (IX), we reason as we did in the corresponding cases of (VIII).

□

*Reflexivity* is the only structural rule that does not hold unrestrictedly in *I*_Ψ_ (the proof is a slight variant of the V-Curry derivation sketched in the introduction).

### **Lemma 8**

*I*_Ψ_ *cannot contain all the instances of*

$$\begin{array}{@{}rcl@{}} \textup{(Ref)} ~~~~~~~~~~~~~~~~~~~~~~~~~~~~~~~~~~~~~~~~~~~~~~~~~~~\varphi \Rightarrow \varphi\kern30pc \end{array} $$

*for* *φ* *an arbitrary* $\mathcal {L}_{V}$ *-sentence.*

Lemma 8 shows also that dropping reflexivity is ‘best possible’: we have a single structural rule that cannot be consistently accepted.^24^ *I*_Ψ_, however, features a weaker form of reflexivity, which follows immediately from the weakening lemma. For every $\varphi \in \mathcal {L}_{V}$, we can always find Γ and $ {\Delta } \subseteq \mathsf {Sent}_{\mathcal {L}_{V}}$ such that ${\Gamma }, \varphi \Rightarrow \varphi , {\Delta } \in \mathsf {I}_{\Psi }$, where Γ and Δ can be taken to be disjoint.

### Naïve Validity in *I*_Ψ_

Several principles for naïve validity (including the (Val-Schema)^+^ formulated and discussed in [9]) are recovered in *I*_Ψ_, in the sense made precise by the following result.

#### **Lemma 9**

*For every* $\varphi , \psi \in \mathcal {L}_{V}$, *and every* ${\Gamma }_{0}, {\Gamma }_{1}, {\Delta }_{0}, {\Delta }_{1} \subseteq \mathsf {Sent}_{\mathcal {L}_{V}}:$

$$\begin{array}{@{}rcl@{}} &&{}(\mathsf{VDm})~\quad\quad\quad\quad\text{ if }~ {\Gamma}_{0} \Rightarrow \varphi, {\Delta}_{0} \text{ is in } \mathsf{I}_{\Psi}\text{ and } {\Gamma}_{1} \Rightarrow \mathsf{Val}(\ulcorner \varphi \urcorner, \ulcorner \psi \urcorner), {\Delta}_{1} \text{ is in } \mathsf{I}_{\Psi}, \\ &&{}\qquad\qquad~~\text{ then } {\Gamma}_{0}, {\Gamma}_{1} \Rightarrow \psi, {\Delta}_{0}, {\Delta}_{1} \text{ is in }\mathsf{I}_{\Psi}. \\ (\mathsf{Val}\text{-}\mathsf{Schema})^{+} \, &&{\Gamma}, \varphi \!\Rightarrow\! \psi, {\Delta} \text{ is in } \mathsf{I}_{\Psi} \text{ if and only if } ~{\Gamma} \!\Rightarrow\! \mathsf{Val}(\ulcorner \varphi \urcorner, \ulcorner \psi \urcorner), {\Delta} \text{ is in } \mathsf{I}_{\Psi}. \end{array} $$

Since (VAl-Schema)^+^ holds in *I*_Ψ_, it is clear that (*VP*) and (VAl-Schema) are recovered in *I*_Ψ_ as well.

(*VDm*) is not to be understood as a ‘weaker version’ of (*VD*), since there are theories that validate (*VD*) but for which (*VDm*) is too strong and yields triviality. The non-transitive approach of Ripley [29] and Cobreros et al. [6] is a case in point: adapting the theory developed there to the validity predicate, we see that (*VD*) holds, while an unrestricted acceptance of (*VDm*) would trivialize the theory. Essentially, this is because (*VDm*) incorporates a form of cut, which is clearly inadmissible in a non-transitive approach.^25^

(*VD*) is the only validity-theoretical principle that does not hold unrestrictedly in *I*_Ψ_. In fact, if *I*_Ψ_ contained all instances of (*VD*), then it would also contain its instance

$$\begin{array}{@{}rcl@{}} \nu, \mathsf{Val}(\ulcorner \nu \urcorner, \ulcorner \bot \urcorner) \Rightarrow \bot \end{array} $$

where *ν* is the V-Curry sentence $\mathsf {Val}(\ulcorner \nu \urcorner , \ulcorner \bot \urcorner )$. The derivation of the V-Curry paradox (outlined in the introduction) would then give us the sequent $\varnothing \Rightarrow \varnothing $ in *I*_Ψ_, against the consistency of *I*_Ψ_.

In Section 3.1 we suggested that a uniform way to avoid V-Curry-driven triviality consists in restricting our acceptance of initial sequents, avoiding the acceptance of Val-sentences that express inferences that we cannot control. Rules of inference, on the other hand, are safe, since the construction of *I*_Ψ_ operates a selection over the sequents that are accepted in the first place. This view sits naturally with a restriction of (*Ref*) and (*VD*) (the only schematic inferences amongst the structural rules and the validity-theoretical principles) and an unrestricted acceptance of weakening, contraction, cut, (*VP*), (*VDm*), and (Val-Schema)^+^. The results 4, 5, 6, 8, and 9, establish that *I*_Ψ_ realizes the solution to the paradoxes described in Section 3.

It is easy to turn *I*_Ψ_ into a proper model of the language $\mathcal {L}_{V}$, as it is standardly done with Kripke fixed points. Let the *extension* of the validity predicate generated by *I*_Ψ_, in symbols *E*_Ψ_, be the set of those pairs of $\mathcal {L}_{V}$-sentences 〈*φ*, *ψ*〉 such that $\varnothing \Rightarrow \mathsf {Val}(\ulcorner \varphi \urcorner , \ulcorner \psi \urcorner )$ is in *I*_Ψ_, and the *anti-extension* of the validity predicate generated by *I*_Ψ_, in symbols **A**_Ψ_, be the set of pairs of $\mathcal {L}_{V}$-sentences 〈*φ*, *ψ*〉 such that $\mathsf {Val}(\ulcorner \varphi \urcorner , \ulcorner \psi \urcorner ) \Rightarrow \varnothing $ is in *I*_Ψ_. The model of $\mathcal {L}_{V}$ naturally associated with *I*_Ψ_, thus, is $(\mathbb {N}, \mathsf {E}_{\Psi }, \mathsf {A}_{\Psi })$, and its evaluation clauses can be read off Definition 4. In particular, $(\mathbb {N}, \mathsf {E}_{\Psi }, \mathsf {A}_{\Psi })$ can be associated with a three-valued semantics, say with values {0,1/2,1}, where the logical vocabulary is interpreted as in strong Kleene semantics (with Val being treated as the strong Kleene conditional), and where ${\Gamma } \Rightarrow {\Delta }$ has a *tolerant-strict* reading, that is whenever every *φ* ∈ Γ has value 1/2 or 1, then there is a *ψ* ∈ Δ with value 1.^26^

We conclude this subsection by noticing that there are close relations between *I*_Ψ_ and the least fixed point of Kripke’s construction for truth (strong Kleene version) from [16]. This can be achieved by defining ¬*φ* and $\mathsf {T}(\ulcorner \varphi \urcorner )$, respectively, as $\mathsf {Val}(\ulcorner \varphi \urcorner , \ulcorner 0=1 \urcorner )$ and $\mathsf {Val}(\ulcorner 0=0 \urcorner , \ulcorner \varphi \urcorner )$, and by constructing the least Kripke fixed point, in the usual way, for the language $\mathcal {L}_{V}$. For every sentence $\varphi \in \mathcal {L}_{V}$ we will have that:

$$\begin{array}{@{}rcl@{}} &&{\kern-1.5pc} \text{if } \varphi \text{ is in the extension of } \mathsf{T} \text{ in the least Kripke's fixed point, then } \varnothing \!\Rightarrow\! \varphi \text{ is in } \mathsf{I}_{\Psi};\\ &&{\kern-1.5pc} \text{if } \varphi \text{ is in the anti-extension of } \mathsf{T} \text{ in the least Kripke's fixed point, then } \varphi \!\Rightarrow\! \varnothing \text{ is in } \mathsf{I}_{\Psi}. \\ (12) \end{array} $$

(12) indicates that Kripke’s least fixed point for truth constitutes a proper fragment of *I*_Ψ_. Clearly, the other direction of (12) does not hold.

### Non-Minimal Fixed Points and Extensions

Lemma 5 shows that every fixed point of Ψ is closed under contraction. However, this is not so for weakening. The fixed point $\{(\varnothing \Rightarrow \mu )\}_{\Psi }$, where *μ* is the sentence $\mathsf {Val}(\ulcorner \mu \urcorner , \ulcorner \mu \urcorner )$, for example, is not closed under weakening (*μ* is the validity-theoretical analogue of the truth-teller). This shortcoming, however, can be easily fixed. Let Ψ^+^ be the monotone operator that results by adding to items (i)-(xi) of Definition 4 the following positive elementary clause as a further disjunct:

- *(xii)* *n* is $({\Gamma }, {\Gamma }^{\prime } \Rightarrow {\Delta }^{\prime }, {\Delta })$, and $({\Gamma } \Rightarrow {\Delta }) \in S$.

Let’s adapt the notation adopted for Ψ to Ψ^+^. The following result is immediate (the first item follows from Lemma 4 and the proof of Proposition 6, and the second by an induction on the build-up of $S_{{\Psi }^{+}}$).

#### **Lemma 10**

- $\mathsf {I}_{\Psi } = \mathsf {I}_{{\Psi }^{+}}$.
- *For every* $S \subseteq \omega $, *S*_Ψ_ *is consistent if and only if* $S_{{\Psi }^{+}}$ *is consistent.*

The properties of *I*_Ψ_ transfer to $\mathsf {I}_{{\Psi }^{+}}$, and the consistency of a fixed point *S*_Ψ_ transfers to the fixed point of Ψ^+^ generated by the same set *S*.^27^ The operator Ψ^+^, however, guarantees closure under weakening, contraction, and cut.^28^

#### **Observation 2**

*For every* $S \subseteq \omega $, *every* $\varphi \in \mathcal {L}_{V}$, *and every* ${\Gamma }, {\Delta } \subseteq \mathsf {Sent}_{\mathcal {L}_{V}}\!\!:$

$$\begin{array}{@{}rcl@{}} \begin{array}{ll} \mathsf{(L}\text{-}\mathsf{Wkn)} \;&\; \text{ If } ~{\Gamma} \!\Rightarrow\! {\Delta} \text{ is in } S_{{\Psi}^{+}}, \text{ then } {\Gamma}, \varphi \Rightarrow {\Delta} \in S_{{\Psi}^{+}}. \\ \mathsf{(R}\text{-}\mathsf{Wkn)} \;&\; \text{ If } ~{\Gamma} \!\Rightarrow\! {\Delta} \text{ is in } S_{{\Psi}^{+}}, \text{ then } {\Gamma} \Rightarrow \varphi, {\Delta} \text{ is in } S_{{\Psi}^{+}}. \\ \mathsf{(L}\text{-}\mathsf{Ctr)} \;&\; \text{ If } ~{\Gamma}, \varphi, \varphi \Rightarrow {\Delta} \text{ is in } S_{{\Psi}^{+}},\text{ then } {\Gamma}, \varphi \Rightarrow {\Delta} \text{ is in } S_{{\Psi}^{+}}. \\ \mathsf{(R}\text{-}\mathsf{Ctr)} \;&\; \text{ If } ~{\Gamma} \!\Rightarrow\! \varphi, \varphi, {\Delta} \text{ is in } S_{{\Psi}^{+}}, \text{ then } {\Gamma} \Rightarrow \varphi, {\Delta} \text{ is in } S_{{\Psi}^{+}}. \\ \mathsf{(Cut)} \;&\; \text{ If } ~{\Gamma} \!\Rightarrow\! \varphi, {\Delta} \text{ is in } S_{{\Psi}^{+}} \text{ and } {\Gamma}, \varphi \Rightarrow {\Delta} \text{ is in } S_{{\Psi}^{+}}, \text{ then } {\Gamma} \Rightarrow {\Delta} \text{ is in } S_{{\Psi}^{+}}. \end{array} \end{array} $$

## From Logical to Grounded Consequence

In this paper, we investigated different combinations and modifications of the principles (*VP*) and (*VD*), and the corresponding notions of consequence. Starting with a restriction of (*VP*) corresponding to the notion of logical consequence, we explored ways of keeping the full (*VP*), thereby restricting (*VD*), carving out a notion of self-applicable consequence grounded in truths and falsities of the base theory. The key findings of the paper are summarized in Table 1.

**Table 1:** Key Findings

Consequence	Key Finding
Logical	(i) Conservativity for all $B \supseteq \mathsf {EA}$
	(ii) *Local* $\mathcal {L}$-embeddability for all $B \supseteq \mathsf {EA}$
Arithmetical	Analysis of Field’s hierarchy via local reflection principles
Grounded	(i) Consistency of (*VP*), (*VDm*), (Val-Schema)^+^
	(ii) Kripke-style theory for Val: development of Ψ
	(iii) Make sense of Val-principles via *I*_Ψ_ and grounded validity

The rules (*VP*) and (*VD*) support a strict reading of *⤙* as logical derivability, and therefore of Val as the class of the logically valid inferences. On this approach, (*VP*), namely (VP_1_) in the formalism of Section 2, is restricted to purely logical inferences, while the full (*VD*) – (VD_1_) in Section 2 – can be consistently kept: from this point of view, we can naturally read (*VD*) as preservation of truth in logically valid arguments. A sub-theory of our theory of logical validity is the theory given by (*VD*) and a suitable version of (*VP*): this theory meets the criterion (suggested by Field in [9]) of giving the same reading to both the meta-theoretic notion expressed by $\vdash $ and to the predicate Val. The theory of logical validity is therefore simple and well-behaved: it’s then natural to study in more depth the corresponding notion of object-linguistic validity by comparing it to the inferential resources of the underlying base theory. Corollary 1 tells us that logical validity does not have any impact on the underlying syntactic structure: for any theory extending a very weak arithmetical system, (*VD*) and the restricted version of (*VP*) do not enable us to prove new syntactic or arithmetical facts. This extends to further principles for logical consequence, like internalized modus ponens, as shown in Corollary 3. These results seem to suggest that ‘deflationary’ approaches to logical consequence, such as Shapiro’s [34], may endorse conservativity requirements for logical validity. This is not to say, however, that the predicate of logical consequence does not play an indispensable expressive role: although the theory of logical validity is uniformly locally interpretable in the base theory, our results *do not* show that it is relatively interpretable in the object theory and therefore, arguably, expressively reducible to it.

As we have seen, the consistency of the theory of logical validity follows from a restriction of (*VP*) to purely logical derivations: consistency, however, is preserved even if we internalize purely arithmetical derivations. This led us, following [9], to investigate a hierarchy of arithmetical consequence predicates. We have shown that every stage in this hierarchy corresponds to a stage of a parallel hierarchy of local reflection principles; as a consequence, the hierarchical notion of validity can be read as iterated arithmetical provability.

The hierarchical approach to consequence suffers from several well-known problems: in particular, there seem to be notions of ‘following from’ that cannot be accounted for in the stratified picture, such as implication. Transcending the hierarchy calls for an unrestricted (*VP*). We have achieved this via a Kripke-style construction, the KV-construction, in which the unrestricted (*VP*) is balanced by a rule form of (*VD*). The least fixed point of the KV-construction, *I*_Ψ_, embodies a notion that, following Kripke’s theory of truth, we might call *grounded validity*, i. e. validity grounded in truths and falsities of the base language, in our case arithmetical truths and falsities.^29^

The main intuition behind grounded validity is that *first* we have inferences involving non-semantic facts, which we can then combine and iterate to express more complex inferences, crucially including nested occurrences of the consequence relation. At the fixed point *I*_Ψ_, the process of generating more and more acceptable inferences reaches a halt: the set *I*_Ψ_ realizes in full the idea of iterating arbitrarily the grounded consequences, and of expressing them in the object-language. To see this, let *F*_Ψ_ be the set of $\mathcal {L}_{V}$-sentences such that *φ* is in *F*_Ψ_ if and only if $\varnothing \Rightarrow \varphi $ is in *I*_Ψ_. It is immediate to see that, thanks to the fixed-point property of *I*_Ψ_, for all sentences *φ*, *ψ* we have that:

$$\begin{array}{@{}rcl@{}} (13){\kern3pc} \mathsf{Val}(\ulcorner \varphi \urcorner, \ulcorner \psi \urcorner) \in \mathsf{F}_{\Psi} \text{ if and only if } \mathsf{Val}(\ulcorner \varnothing \urcorner, \ulcorner \mathsf{Val}(\ulcorner \varphi \urcorner, \ulcorner \psi \urcorner) \urcorner) \in \mathsf{F}_{\Psi}. \end{array} $$

(13) follows immediately by Lemma 9, that shows that (Val-Schema)^+^ holds unrestrictedly in *I*_Ψ_. In his [9], Field rejects (Val-Schema)^+^ on the grounds of an example that, informally, reads thus:

$$\begin{array}{@{}rcl@{}} &&\text{It follows } \,\text{from `snow is white' that}\\ (14){\kern5pc}\\ &&\qquad\,\,\qquad\text{it follows from `grass is green' that `snow is white'. } \end{array} $$

If ‘follows from’ is intended as logical consequence, (14) clearly does not hold. However, (14), and more generally (Val-Schema)^+^, cease to be troubling if we adopt the grounded consequence reading: since snow is white, this truth about the non-semantic vocabulary grounds and justifies the consequence expressed in (14).^30^

Desirable features such as that expressed in (13) come at a cost: we cannot consistently accept all sequents of the form $\varphi \Rightarrow \varphi $ in *I*_Ψ_. As a consequence, not all sentences of the form $\mathsf {Val}(\ulcorner \varphi \urcorner , \ulcorner \varphi \urcorner )$ are in *F*_Ψ_. However, this restriction is not so implausible in the context of a grounded consequence relation. Some sequents of the form $\varphi \Rightarrow \varphi $, in fact, are to be rejected because they are *ungrounded*. A paradigmatic case of failure of (*Ref*) involves the V-Curry sentence itself: we do not have $\nu \Rightarrow \nu $ in *I*_Ψ_. In other words, we do not accept that

$$\begin{array}{@{}rcl@{}} \text{ from the fact that (from this sentence it follows that } 0=1), \\ (15){\kern26pc}\\ \text{ it follows that (from this sentence it follows that } 0=1). \end{array} $$

If we only accept grounded Val-sentences, we want to *unpack* the ‘it follows’ used in (15), to see from where it derives. Given the ungrounded nature of *ν*, such unpacking does not lead us to non-semantic inferences, but to an endless, circular regress. Cases such as (15) are the only kind of sequents of the form $\varphi \Rightarrow \varphi $ that are not in *I*_Ψ_, which makes the restriction of reflexivity less drastic.^31^ Similarly, ungrounded instances of (*VD*) are not in *I*_Ψ_, and this restriction is justified as in the case of reflexivity.

### Future Work

We conclude by sketching some directions for further work. In the context of logical validity, the main open problem is the question of the global interpretability and, most importantly, of the $\mathcal {L}$-embedding of the theory of logical validity in the base theory. As for grounded consequence, the construction described by *I*_Ψ_ can be turned into an axiomatic theory. It would then be natural to study the relationships between this theory and the class of models extending $(\mathbb {N}, \mathsf {E}_{\Psi }, \mathsf {A}_{\Psi })$. Finally, it would be interesting to relate irreflexive validity with paracomplete theories of truth and validity.^32^

## Footnotes

1 To emphasize even more the analogy with truth, we note that an object-linguistic predicate for consequence is needed for the same purposes that motivate a truth predicate, such as blind ascriptions (‘all the derivations made this morning in the Logic lecture are valid’) or generalizations.

2 Suppose in fact that (*V**P*) is logically valid, that there is a logical derivation of *φ* *⤙* *ψ*, and that *f* is a function in the language from names of sentences to terms that do not name sentences. Then applying (*V**P*) and uniform substitutivity we conclude that $\mathsf {Val}(f(\ulcorner \varphi \urcorner ), f(\ulcorner \psi \urcorner ))$ comes out as logically derivable, which is absurd. This is clearly remarked in [7].

3 Ketland’s results will turn out to be special cases of our findings.

4 See [28].

5 For a quirk of terminology, ‘reflexive’ will be employed in two different senses: (i) as referred to arithmetical systems that prove the consistency of all their finite subsystems, and (ii) as referred to theories that satisfy the structural rule of reflexivity. We apologize for the possible, but unavoidable, confusion.

6 It is also clear that the paradox can be derived by avoiding any syntactic/arithmetical assumption. To see this, one can employ a familiar trick due to Montague [21]. A finite formulation of the axioms of a finitely axiomatizable *T* (see below for the choice of *T*) can be pushed into the instance of the diagonal lemma needed in the derivation of the paradox. Starting with the instance

$$\nu \leftrightarrow \mathsf{Val}(\ulcorner \nu\land { A}\urcorner,\bot) $$

of the diagonal lemma, where *A* is a finite reaxiomatization of *T*, one easily obtains a version of the paradox that does not rely on the assumption of the logical validity of the underlying arithmetical theory but only of the principles for logical consequence.

7 For more details on *E**A* and elementary functions the reader may consult [3, 33]. The axioms of *E**A* are the universal closures of the following:

$$\begin{array}{llllll} \mathsf{EA1}\quad& 0\neq \mathsf{S}x \\ \mathsf{EA2}\quad& \mathsf{S}x=\mathsf{S}y\rightarrow x=y\\ \mathsf{EA3}\quad& x+0=x\\ \mathsf{EA4}\quad& x+\mathsf{S}y=\mathsf{S}(x+y)\\ \mathsf{EA5}\quad& x\times 0=0\\ \end{array} \hspace{1cm} \begin{array}{llllll} \mathsf{EA6}\quad& x\times \mathsf{S}y=(x\times y)+x\\ \mathsf{EA7}\quad& \mathsf{exp}(0)=1\\ \mathsf{EA8}\quad& \mathsf{exp}(\mathsf{S}x)=\mathsf{exp}(x)+\mathsf{exp}(x)\\ \mathsf{EA9}\quad& x\leq 0 \,\leftrightarrow\,x=0\\ \mathsf{EA10}\quad& x\leq \mathsf{S}y\,\leftrightarrow\,(x\leq y\vee x=\mathsf{S} y) \end{array} $$

In addition, *E**A* features the principle of induction for *elementary formulas* *φ*(*x*):

8 The notion of relative interpretation goes back to Tarski et al. [36]. Although it is a syntactic notion, it has a natural semantic counterpart: if *U* is interpretable in *W*, in any model of *W* we can construct an internal model of *U*.

9 Feferman’s theorem on the interpretability of inconsistency (see [38]) represents a separating example between relative interpretation and $\mathcal {L}$-embeddings. Let *T* be *ω*-consistent: by Feferman’s theorem, *T* + ¬*C**o**n*(*T*) is interpretable in *T*, but it cannot be $\mathcal {L}$-embeddable in *T* because $\mathcal {L}$-embeddability clearly preserves *ω*-inconsistency. For a study of how to separate $\mathcal {L}$-embeddings from stronger notions of equivalence, see [26].

10 We refer to [13, Section 5.3] for motivation andto [33, Section 2.6.1] for a proof of the theorem in *E**A*.

11 This is the standard objection against classical theories such as a version of the well-known theory Kripke-Feferman *K**F*. See [8, 13].

12 The same proof applies to $T^{\mathsf {V}}\!\!\upharpoonright $.

13 The theorem states that if *V* is reflexive and any finite $U_{0}\subseteq U$ is interpretable in *V*, also *U* is interpretable in *V* (see [12, Secton 3]).

14 By employing the sequence encoding sketched above, one can estimate for the translated proof:$|\mathcal {D}^{\mathfrak {b}}_{j}|\leq G(n)$, where$G(n)=\underbrace {\langle n(n+1){2^{n}},\ldots ,n(n+1){2^{n}}\rangle }_{n\text {-times}}=\left (n(n+1){2^{n}}+1\right ){2^{(n(n+1){2^{n}})}}$

15 We adapt to the present setting a more general strategy suggested to us by Albert Visser.

16 In the definition of *C*_*n*_, (*x*)_*y*_ = *z* is an elementary functional expression corresponding to the projection of the *y*^*th*^-element of*x*.

17 For a study of the asymmetry between finitely axiomatized and reflexive theories in the context of theories of truth, see [25].

18 In particular, *O**R* is an elementary set of ordinal codes and $\prec $ an elementary relation isomorphic to the usual well-ordering of ordinals up to Γ_0_.

19 It is nonetheless possible to develop axiomatic theories that are adequate for these models and therefore avoid any arbitrariness in choosing a natural halting point for progressions of theories of stratified validity.

20 Some substructural theories compatible with Beall and Murzi’s principles are available in the literature: the *non-contractive* theory in [41] (see also [5]) and the *non-transitive* approach of [29] and [6] (see also [37]) support both (*V**P*) and (*V**D*), and block the derivation of ⊥. Strictly speaking, these are theories of naïve truth that feature a conditional obeying conditional versions of (*V**P*) and (*V**D*), but they can be easily adapted to naïve validity.

21 For the theory of inductive definitions, see [22]. Non-reflexive approaches to paradoxes did not receive an extensive attention in the literature: some works on the topic include [10, 11, 31, 32].

22 For a strengthening of Meadows’ approach, see [35].

23 Consistency is typically defined as the absence of a contradiction, but our definition is equivalent to that. We could introduce a connective ¬, interpreting ¬*φ* as $\mathsf {Val}(\ulcorner \varphi \urcorner , \ulcorner 0=1 \urcorner )$, and show that the classical rules for negation hold for the so-defined ¬ in *I*_Ψ_. Then, it would be easy to show that $\varnothing \Rightarrow \varnothing \notin \mathsf {I}_{\Psi }$ if and only if there is no $\mathcal {L}_{V}$-sentence *φ* s. t. $\varnothing \Rightarrow \varphi \wedge \neg \varphi \in \mathsf {I}_{\Psi }$. We note that the resulting negation is weaker than classical negation, and indeed even weaker than intuitionistic negation (not al the *ex falso* sequents $\varphi \wedge \neg \varphi \Rightarrow \bot $ are in *I*_Ψ_, although the corresponding rule of inference is admissible in *I*_Ψ_).

24 [32] has recently remarked that a similar restriction of reflexivity in the context of rules for naïve comprehension can avoid paradoxes and, at the same time, make both cut and contraction admissible.

25 The choice between (*V**D*) and (*V**D**m*) reflects an ongoing debate in the truth-theoretical literature (especially concerning substructural theories of truth) on the ‘correct’ form of *modus ponens* (see [42] and [30]). Two of the main contenders are analogous to (*V**D*) and (*V**D**m*):

$$\varphi, \varphi \rightarrow \psi \vdash \psi \quad\text{and} \quad \text{from } {\Gamma} \vdash \varphi \text{ and } {\Delta} \vdash \varphi \rightarrow \psi \text{ infer } {\Gamma}, {\Delta} \vdash \psi. $$

26 We are grateful to Paul Egré, Francesco Paoli, and Robert van Rooij for suggesting this point to us. Tolerant-strict consequence is also employed in [20].

27 We did not use Ψ^+^ in the first place in order to simplify the proofs of the properties of *I*_Ψ_: this has no practical effects, however, since *I*_Ψ_ and $\mathsf {I}_{{\Psi }^{+}}$ are identical.

28 This result improves on the structural rules recovered by Meadows in [20].

29 See [16], p. 694 and p. 701. For an analysis of Kripkean groundedness, see [40]. For arguments for Kripkean grounded truth, see [17, 19], and [4].

30 Grounded validity is clearly distinct from analytical validity, i. e. validity based on analytical truths and falsities. In fact, it is possible to start our construction from non-analytic claims, e. g. about snow being white and grass being green. Thanks to Andreas Fjellstad for bringing this point to our attention.

31 The standard counterpart of of reflexivity in natural deduction is the rule of assumption (see [24], Section 1. 3). If we accept a grounded picture of consequence, we can safely assume all the sentences of the base language, and apply all rules of inferences to them: the only sentences we cannot assume are the ungrounded ones, in line with our conception of consequence. Moreover, it is easy to see that the notion of consequence captured by *I*_Ψ_ includes all the logical consequences grounded in the base language. Many thanks to Kit Fine, Lionel Shapiro, and Luca Tranchini for useful discussions on these points.

32 The latter research question has also been suggested in [10].
